# Effect of Behaviorally Designed Gamification With Social Incentives on Lifestyle Modification Among Adults With Uncontrolled Diabetes: A Randomized Clinical Trial

**DOI:** 10.1001/jamanetworkopen.2021.10255

**Published:** 2021-05-24

**Authors:** Mitesh S. Patel, Dylan S. Small, Joseph D. Harrison, Victoria Hilbert, Michael P. Fortunato, Ai Leen Oon, Charles A. L. Rareshide, Kevin G. Volpp

**Affiliations:** 1 Department of Medicine, Perelman School of Medicine, University of Pennsylvania, Philadelphia; 2 Wharton School, University of Pennsylvania, Philadelphia; 3 Penn Medicine Nudge Unit, University of Pennsylvania, Philadelphia; 4 Department of Medicine, University of Pennsylvania, Philadelphia

## Abstract

**Importance:**

Gamification is increasingly being used to promote healthy behaviors. However, it has not been well tested among patients with chronic conditions and over longer durations.

**Objective:**

To test the effectiveness of behaviorally designed gamification interventions to enhance support, collaboration, or competition to promote physical activity and weight loss among adults with uncontrolled type 2 diabetes.

**Design, Setting, and Participants:**

A 4-arm randomized clinical trial with a 1-year intervention was conducted from January 23, 2017, to January 27, 2020, with remotely monitored intervention. Analyses were conducted between February 10 and October 6, 2020. Participants included 361 adults with type 2 diabetes with hemoglobin A_1c_ levels greater than or equal to 8% and body mass index greater than or equal to 25.

**Interventions:**

All participants received a wearable device, smart weight scale, and laboratory testing. Participants in the control group received feedback from their devices but no other interventions. Participants in the gamification arms conducted goal setting and were entered into a 1-year game designed using insights from behavioral economics with points and levels for achieving step goals and weight loss targets. The game varied by trial arm to promote either support, collaboration, or competition.

**Main Outcomes and Measures:**

Co-primary outcomes included daily step count, weight, and hemoglobin A_1c_ level. Secondary outcome was low-density lipoprotein cholesterol level. Intention-to-treat analysis was used.

**Results:**

Participants had a mean (SD) age of 52.5 (10.1) years; hemoglobin A_1c_ level, 9.6% (1.6%); daily steps, 4632 (2523); weight, 107.4 kg (20.8 kg); and body mass index, 37.1 (6.6). Of the 361 participants, 202 (56.0%) were women, 143 (39.6%) were White, and 185 (51.2%) were Black; with 87 (24.1%) randomized to control; 92 (25.4%) randomized to gamification with support and intervention; 95 (26.3%) randomized to gamification with collaboration; and 87 (24.1%) randomized to gamification with competition. Compared with the control group over 1 year, there was a significant increase in mean daily steps from baseline among participants receiving gamification with support (adjusted difference relative to control group, 503 steps; 95% CI, 103 to 903 steps; *P* = .01) and competition (606 steps; 95% CI, 201 to 1011 steps; *P* = .003) but not collaboration (280 steps; 95% CI, −115 to 674 steps; *P* = .16). All trial arms had significant reductions in weight and hemoglobin A_1c_ levels from baseline, but there were no significant differences between any of the intervention arms and the control arm. There was only 1 adverse event reported that may have been related to the trial (arthritic knee pain).

**Conclusions and Relevance:**

Among adults with uncontrolled type 2 diabetes, a behaviorally designed gamification intervention in this randomized clinical trial significantly increased physical activity over a 1-year period when designed to enhance either support or competition but not collaboration. No differences between intervention and control groups were found for other outcomes.

**Trial Registration:**

ClinicalTrials.gov Identifier: NCT02961192

## Key Points

**Question:**

Can gamification designed to incorporate behavioral insights and social incentives promote lifestyle modification over a 1-year period among adults with uncontrolled type 2 diabetes?

**Findings:**

In this randomized clinical trial of 361 adults with overweight or obesity and uncontrolled type 2 diabetes, gamification interventions designed to enhance support or competition each significantly increased physical activity relative to controls during the 1-year intervention, but gamification with collaboration did not significantly change physical activity relative to controls. All study arms had significant reductions in weight and hemoglobin A_1c_ levels from baseline, but none of the gamification interventions resulted in significant differences in these outcomes relative to controls.

**Meaning:**

Gamification designed to incorporate behavioral insights and enhance either competition or support resulted in increases in physical activity in this randomized clinical trial but may need to be combined with other interventions to promote weight loss or changes in glycemic control.

## Teaser

This randomized clinical trial examines the effectiveness of behaviorally designed gamification to promote lifestyle change among adults with uncontrolled type 2 diabetes.

## Introduction

In the US, there are more than 34 million adults with type 1 or type 2 diabetes, representing 13% of the population.^1^ Lifestyle modification, including physical activity and weight loss, have been demonstrated to improve diabetes control.^1,2,3,4,5,6,7^ However, sustaining changes to these healthy behaviors can be challenging for many patients.^8,9^

Gamification is the use of game design elements, such as points and levels, that has increasingly been used to engage individuals in health promotion efforts.^10,11,12^ Many employers commonly use gamification in workplace wellness programs,^13,14,15^ and game design elements exist in about two-thirds of the most popular health and fitness mobile applications.^16^ Although gamification is used widely, designs often do not fully leverage insights from fields such as behavioral economics that could better address predictable barriers to behavior change.^10,12,16,17^

Two previous randomized clinical trials^18,19^ demonstrated that behavioral economic principles and social incentives could be embedded within the design of a gamification intervention to significantly increase physical activity during 3- and 6-month interventions, with effects sustained during 3-month follow-up periods after the interventions were stopped. A pilot study was conducted to test these approaches for promoting weight loss.^20^ However, these studies were relatively short and did not target patients with chronic disease.

In this study, our objective was to conduct a longer-term randomized clinical trial to test the effectiveness of gamification interventions that incorporated behavioral insights and used supportive, collaborative, or competitive social incentives to promote physical activity and weight loss among adults with overweight or obesity and uncontrolled type 2 diabetes. Patients were remotely monitored for 1 year by using wearable devices, a smart weight scale, and an automated technology platform.^21^

## Methods

### Trial Design

 Influencing Diabetics to Adapt Behaviors Related to Exercise and Weight by Enhancing Social Incentives (iDiabetes) was a randomized clinical trial testing behaviorally designed gamification with support, collaboration, or competition relative to controls to promote physical activity and weight loss among adults with uncontrolled type 2 diabetes. The trial was conducted between January 23, 2017, and January 27, 2020, and consisted of a 2-week run-in period, a baseline in-person visit, and then a 1-year remotely monitored intervention. Participants provided informed consent and received equipment for use during the study and financial compensation. Analyses were conducted between February 10 and October 6, 2020. Details of the study design and protocol have been published previously.^22^ The trial protocol (Supplement 1) was approved by the University of Pennsylvania Institutional Review Board. This trial followed the Consolidated Standards of Reporting Trials (CONSORT) reporting guideline.

The study was conducted using Way to Health,^21^ a research technology platform at the University of Pennsylvania used previously for remote monitoring and behavioral interventions.^18,19,23,24,25,26,27^ Participants used the study website to create an account, provide informed consent online, and complete baseline and validated survey assessments. Each participant selected whether to receive regular study communications by email, text message, or both. Eligible participants had laboratory testing and were mailed a wrist-worn wearable device (Withings Activite Steel; Withings Inc) that lasts about 6 months without charging, along with a replacement battery. Participants used a smartphone application (Withings HealthMate) to connect their devices to the Way to Health platform for remote data collection and used the devices to track daily step counts. Prior work has demonstrated that these types of devices are accurate for tracking step counts^28^ and have been used successfully in earlier studies.^13,18,19,23^ After completing the 2-week run-in phase, eligible participants had an in-person visit to receive a smart weight scale (Withings Body) and conduct their baseline weigh-in. Participants received $25 for completing baseline laboratory testing and $25 for enrolling in the trial. At 6 and 12 months, participants received $50 to obtain laboratory tests (hemoglobin A_1c_ [HbA_1c_] [to convert to proportion of total hemoglobin, multiply by 0.01] and low-density lipoprotein cholesterol [LDL-C]) and conduct a virtual weigh-in at home via FaceTime (Apple Inc) or Skype (Microsoft Corp), as conducted in a prior study.^20^ At 12 months, participants completed a survey on their experience in the study.

### Participants

Potential participants were identified from the electronic health records at Penn Medicine and invited to learn more about the study online. The study team conducted outreach to approximately 13 500 patients by email, mailed letter, or phone call. Recruitment occurred from January 23, 2017, to January 7, 2019. Study accrual progressed slower than anticipated and led to the body mass index (BMI) threshold being decreased from 30 to 25 (calculated as weight in kilograms divided by height in meters squared). This lowered lack of interest may have occurred because these patients were a less motivated group, and our primary recruitment strategy was a cold email from the study team.

Participants were eligible for the program if they were between ages 18 and 70 years; able to read and provide informed consent; had a diagnosis of type 2 diabetes with the most recent HbA_1c_ level greater than or equal to 8.0%; and, within the past 90 days, had a self-reported BMI greater than or equal to 25; and owned a smartphone or tablet compatible with the wearable device and a smart weight scale. Individuals were excluded if there was a condition that made their participation infeasible (eg, inability to provide informed consent or inability to speak, read, or write in the English language); if there was a condition that made participation unsafe (eg, pregnancy, previous diagnosis of an eating disorder, or history of unsafe weight loss practices); if they were already enrolled in another study targeting physical activity, weight loss, or glycemic control; or if any other medical conditions or reasons prohibited the individual from participating in the 1-year trial. Participants were informed that, depending on intervention arm assignment, they may be grouped with 2 other participants whom they did not previously know (n = 357). Because earlier work indicated that these interventions may be more effective if participants know each other,^20,29^ we encouraged participants to find a family member or friend to enroll with them as a group of 2 or 3 persons (n = 4).

### Baseline Assessment

Participants who were eligible after laboratory testing were mailed the wearable device. After the wearable device was set up and connected to the study, the participant was asked to become accustomed to wearing the device for several weeks. During this run-in period, a baseline step count was estimated using the second week of data—a method used in previous work.^18,19,23^ The first week of data was ignored to diminish the potential upward bias of the estimate from higher activity during initial device use. If less than 4 days of data were available during the second week (n = 15), the participant was contacted to inquire about any device issues and the run-in period was extended until at least 4 days of data were captured. After establishing a baseline step count, participants had an in-person visit with the study team. At the visit, participants were given the smart weight scale and conducted a baseline weigh-in.

### Randomization

After establishing a baseline weight, participants were randomized electronically using block sizes of 4 groups with 3 participants per group. Participants were randomized in groups of 3 because of the design of the collaborative and competitive gamification interventions. All investigators, statisticians, and data analysts were blinded to arm assignments until the study and analysis were completed.

### Interventions

During the in-person visit, all participants received education on the importance of diet and physical activity for weight loss and glycemic control using recommendations from the Centers for Disease Control and Prevention.^30^ Participants in the control arm received regular feedback and goal setting from the devices and smartphone application but received no other interventions. Participants randomly assigned to 1 of the 3 gamification interventions conducted goal setting during the in-person visit including selecting an HbA_1c_ reduction goal (1.5%, 2%, or 2.5%), a weight loss goal (6%, 7%, or 8%), and a step count increase (33%, 40%, 50%, or any goal ≥1500 steps above baseline). These options were based on prior work^18,19,20^; participants were told to strive for these goals during the first 6 months and maintain them through 12 months. Participants were given a weekly weight target (about 1 lb or less) that gradually declined to the goal by 6 months. If a weekly goal was not achieved, the target remained the same for the following week. Similar to earlier work,^19^ step targets increased gradually over 4 weeks.

Participants in the intervention arms were entered into a game with points and levels that ran automatically (participants did not have to actively play the game—just strive for their goals) and provided a daily notification on their progress. The design was based on previous work that incorporated principles from behavioral economics.^18,19,20^

First, participants in the gamification arms signed a precommitment pledge to strive to achieve their goals during the 1-year trial.^31,32^ In earlier work,^18^ step goal targets began immediately, which was challenging for some participants. Therefore, in this trial, participants had a ramp-up period during the first 4 weeks in which daily step goal targets increased by 25% per week from baseline to the goal. Participants were asked to strive for this step goal for the rest of the trial but could change the goal at any point as long as it was within the options provided.

Second, every Monday, the participant received 70 points (10 for each day of the week). If the participant did not weigh in on the prior day, they lost 10 points from their balance. This practice leverages prospect theory,^33^ which has demonstrated that loss framing is more effective at motivating behavior change than gain framing.^25,33^

Third, at the end of each week, participants could move up or down levels (from lowest to highest: blue, bronze, silver, gold, and platinum). This design creates achievable goal gradients, a sense of social status, and progression through the game. If at the end of each week the participant had at least 40 points, achieved their weekly weight target, and averaged at or above their daily step goal, they would move up a level.

Fourth, we leveraged the “fresh start effect,” which is the tendency for aspirational behavior around temporal landmarks, such as the beginning of the year, month, or week.^34^ Similar to prior work,^18,19,20^ participants started each week with a fresh set of 70 points. To help re-engage participants who were struggling to meet their goals at months 3, 6, and 9 (defined as being in the blue or bronze levels of the game), the study team called them to inquire about their progress in the study, reset them to the silver level, and offered them the opportunity to readjust their goals based on their initial options.

Fifth, similar to an earlier study,^20^ the participants’ primary care physician was mailed a monthly report with data on their change in step counts, weight, HbA_1c_ level, and LDL-C level as a way of increasing social accountability.^35^ A copy of this letter was also sent to the participant by email each month.

Sixth, the game varied based on the social incentive arm as follows. In the support arm, participants were asked to identify a family member or friend who would be a support sponsor and be emailed a weekly report on the participant’s performance in the game over the past week (goal attainment, points, and level) and their targets for the upcoming week (step counts, weight, and points). This sponsor was sent a message at the start of the trial to do their best to support the participant in their progress during the interventions.

In the collaboration arm, participants were placed into a team of 3 total participants. These individuals typically did not know each other before the study but were introduced to each other by email. Each day one of the members of the group was randomly selected to represent their team for that day, and that information was shared with the entire group. If the selected participant weighed in on the previous day, the team kept their points. If he or she did not, then the entire team lost 10 points. In this design, each person is accountable to the others on the team, and this strategy was intended to induce a collaborative effort to meet their daily goals. The entire team moved up a level only if the team had at least 40 points by the end of the week.

In the competition arm, participants were placed into a group of 3 total participants. These individuals typically did not know each other before the study but were introduced to each other by email. At the end of each week, the participants received an email with a leaderboard that ranked them on their cumulative points in the study thus far and displayed their level. This feedback was intended to encourage participants to compete for the top spot among the group.

### Outcome Measures

The primary outcomes were change in daily number of steps, weight, and HbA_1c_ level from baseline to the end of the 12-month trial. Secondary outcomes included change in LDL-C level from baseline to the end of the 12-month trial as well as change in daily number of steps, weight, HbA_1c_ level, and LDL-C level from baseline to 6 months.

### Statistical Analysis

We estimated that a sample size of 360 participants (90 per arm) would provide at least 80% power using a conservative Bonferroni adjustment of the type I error rate with a 2-sided α level of .017 and accounting for a dropout rate of 10% to detect (1) a 1100-step change in physical activity, with an SD of 2000 steps; (2) a 6-lb change in weight, with an SD of 10 lb; and (3) a 0.8% change in HbA_1c_ level, with an SD of 1.5%. These values were based on earlier work.^18,20,36,37^

All randomly assigned participants were included in the intention-to-treat analysis. For each patient on each day of the study (participant-day level), the number of steps achieved was obtained as a continuous variable. Weight, HbA_1c_ level, and LDL-C level were collected at 6 and 12 months as continuous variables. Missing data rates are available (eTable 1 and eTable 2 in Supplement 2) and were similar to those in earlier work.^18,19,20,38^

For the prespecified main analysis, we used multiple imputation for missing data as in previous studies.^18,23,24,25,26,27^ Five imputations were conducted using the mice package in R, version 3.4.0 (R Foundation for Statistical Computing), which allows for participant random effects with this data structure.^39^ The following factors associated with missing data were included: trial arm, week of study, calendar month, baseline outcome measure, age, sex, race/ethnicity, educational level, marital status, annual household income, self-reported health, and the Prochaska stage of change^40^ for trying to lose weight. Results were combined using Rubin’s standard rules.^41^ This imputation approach has been used in previous work.^18,23^ Sensitivity analyses were conducted using collected data without multiple imputation.

Similar to previous work,^18,23^ adjusted analyses fit generalized mixed-effects models with a random intercept and participant random effects; in the physical activity models, adjusted analyses were fit to account for the repeated measures of daily step counts. The adjusted models included the baseline measure and fixed effects for calendar month and trial arm. Differences in the outcome measure between arms were obtained using the least-squared means command. In a secondary analysis, we examined the trajectory of change in daily step counts within each arm by comparing the last 6 months of the trial with the first 6 months, adjusted for baseline step count and calendar month fixed effects.

## Results

**Figure 1.**
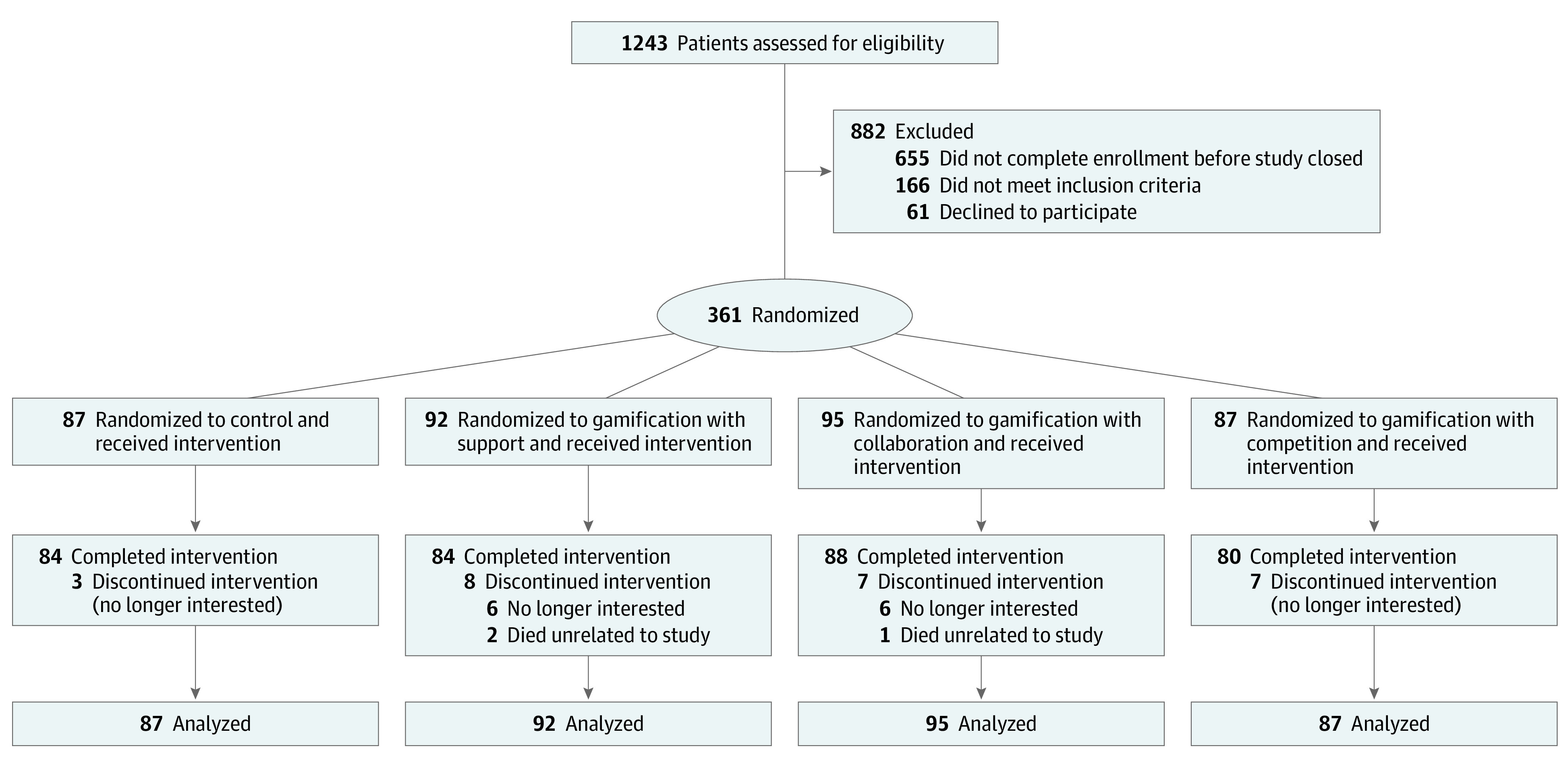
Consolidated Standards of Reporting Trials Flow Diagram Participants in all trial arms received a wearable device and smart weight scale and established baseline measures. Participants in the control group received regular feedback from the wearable device, weight scale, and its smartphone application but no other interventions. Participants in the gamification arms set goals for daily steps, weight loss, and hemoglobin A_1c_ level targets and then were entered into 1 of 3 gamification interventions that ran automatically for 1 year.

In this trial, 361 participants were randomized (Figure 1). Two groups (n = 4 participants) comprised individuals who knew each other, and the other groups included 357 participants who did not know each other. Participants had a mean (SD) age of 52.5 (10.1) years; HbA_1c_ level, 9.6% (1.6%); number of daily steps, 4632 (2523); weight, 107.4 kg (20.8 kg); and BMI, 37.1 (6.6). Of the 361 participants, 202 (56.0%) were women, 159 (44.0%) were men, 143 (39.6%) were White, and 185 (51.2%) were Black; with 87 (24.1%) randomized to control; 92 (25.4%) randomized to gamification with support and intervention; 95 (26.3%) randomized to gamification with collaboration; and 87 (24.1%) randomized to gamification with competition. . Baseline participant characteristics were well balanced across the trial arms (Table 1).

**Table 1.** Characteristics of Study Participants

Characteristic	No. (%)			
	Control (n = 87)	Gamification		
		With support (n = 92)	With collaboration (n = 95)	With competition (n = 87)
**Sociodemographic**				
Age, mean (SD), y	53.4 (10.6)	52.8 (10.4)	52.5 (9.1)	51.3 (10.6)
Sex				
Female	48 (55.2)	56 (60.9)	57 (60.0)	41 (47.1)
Male	39 (44.8)	36 (39.1)	38 (40.0)	46 (52.9)
Race/ethnicity				
White non-Hispanic	35 (40.2)	30 (32.6)	37 (38.9)	41 (47.1)
Black non-Hispanic	45 (51.7)	52 (56.5)	50 (52.6)	38 (43.7)
Other^a^	7 (8.0)	10 (10.9)	8 (8.4)	8 (9.2)
Educational level				
Some high school	2 (2.3)	5 (5.4)	1 (1.1)	2 (2.3)
High school graduate	7 (8.0)	6 (6.5)	13 (13.7)	10 (11.5)
Some college or specialized training	34 (39.1)	32 (34.8)	37 (38.9)	32 (36.8)
College graduate	44 (50.6)	49 (53.3)	44 (46.3)	43 (49.4)
Annual household income, $				
<30 000	14 (16.1)	19 (20.7)	20 (21.1)	15 (17.2)
30 000-60 000	23 (26.4)	19 (20.7)	18 (18.9)	20 (23.0)
60 001-100 000	13 (14.9)	18 (19.6)	24 (25.3)	15 (17.2)
>100 000	22 (25.3)	19 (20.7)	15 (15.8)	25 (28.7)
Declined to answer	15 (17.2)	17 (18.5)	18 (18.9)	12 (13.8)
Marital status				
Single	29 (33.3)	25 (27.2)	32 (33.7)	38 (43.7)
Married	47 (54.0)	55 (59.8)	52 (54.7)	37 (42.5)
Other	11 (12.6)	12 (13.0)	11 (11.6)	12 (13.8)
**Baseline measures**				
Daily step count, mean (SD)	4410 (2263)	4353 (2439)	4122 (2817)	4681 (2893)
Weight, mean (SD), kg	106.8 (22.5)	103.4 (18.2)	110.8 (21.4)	108.3 (20.7)
BMI, mean (SD)	37.2 (6.7)	36.7 (6.3)	38.0 (6.6)	36.6 (6.9)
Hemoglobin A_1c_, mean (SD), %	9.5 (1.5)	9.5 (1.5)	9.9 (1.7)	9.7 (1.6)
LDL-C, mean (SD), mg/dL	99.9 (31.2)	101.7 (41.8)	97.9 (32.4)	90.0 (38.7)
Charlson Comorbidity Index, median (IQR)	2 (1-3)	2 (1-4)	2 (1-4)	2 (1-4)
**Self-reported measures**				
Currently smoking	4 (4.6)	2 (2.2)	6 (6.3)	4 (4.6)
Currently using other tobacco products	5 (5.7)	4 (4.3)	5 (5.3)	7 (8.0)
Health status				
Excellent	0	0	0	0
Very good	12 (13.8)	6 (6.5)	3 (3.2)	4 (4.6)
Good	37 (42.5)	42 (45.7)	40 (42.1)	33 (37.9)
Fair	32 (36.8)	40 (43.5)	45 (47.4)	40 (46.0)
Poor	6 (6.9)	4 (4.3)	7 (7.4)	10 (11.5)
Prescribed medications				
Oral diabetes	32 (36.8)	36 (39.1)	43 (45.3)	33 (37.9)
Insulin	65 (74.7)	63 (68.5)	72 (75.8)	61 (70.1)
Statin	38 (43.7)	49 (53.3)	44 (46.3)	48 (55.2)
Prior use of smartphone or wearable to track step counts	47 (54.0)	54 (58.7)	55 (57.9)	46 (52.9)
Prior use of wireless scale to track weight	7 (8.0)	7 (7.6)	6 (6.3)	6 (6.9)

Abbreviations: BMI, body mass index (calculated as weight in kilograms divided by height in meters squared); IQR, interquartile range; LDL-C, low-density lipoprotein cholesterol.

SI conversion factors: To convert hemoglobin A_1c_ to proportion of total hemoglobin, multiply by 0.01; LDL-C to millimoles per liter, multiply by 0.0259.

^a^ Other represents a convenience label without further clarification of categories.

### Physical Activity

**Figure 2.**
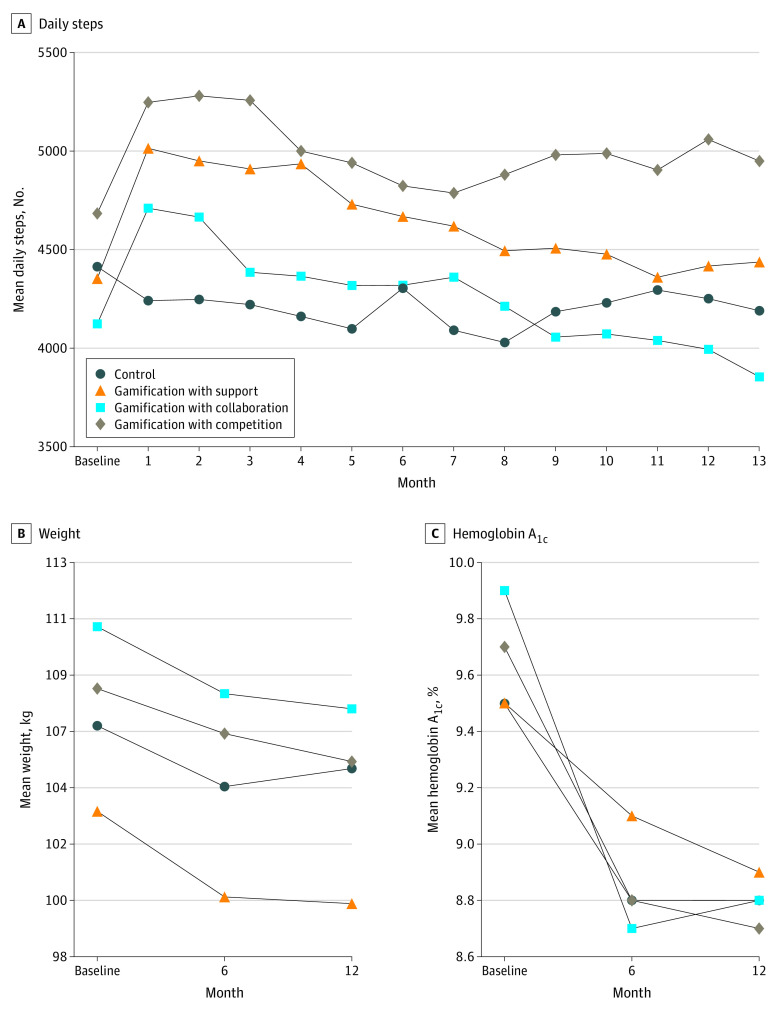
Unadjusted Outcomes Depicted are outcome measures using imputed data as the mean number of daily steps for each arm by month (A); mean weight at baseline, 6 months, and 12 months (B); and mean hemoglobin A_1c_ level at baseline, 6 months, and 12 months. To convert hemoglobin A_1c_ to proportion of total hemoglobin, multiply by 0.01.

The unadjusted mean number of daily steps by month and study arm are depicted in Figure 2A. During the first few months of the trial, mean daily steps decreased from baseline among participants in the control arm but increased among those in the gamification arms. In the control arm, participants had a mean of 4410 steps per day at baseline and a mean of 4221 in month 3; in the gamification with competition arm, participants had a mean of 4681 steps per day at baseline and 5257 in month 3; in the gamification with support arm, the number of steps was 4353 at baseline and 4909 at month 3; and in the gamification with collaboration arm, the number of steps was 4122 at baseline and 4385 at month 3.

In the adjusted models compared with the control group at 1 year, there was a significant increase in mean number of daily steps from baseline among participants in gamification with support (adjusted difference relative to control group, 503 steps; 95% CI, 103 to 903 steps; *P* = .01) and competition (606 steps; 95% CI, 201 to 1011 steps; *P* = .003) but not collaboration (280 steps; 95% CI, −115 to 674 steps; *P* = .16) (Table 2). Results were similar in sensitivity analyses that used collected data without multiple imputation (eTable 3 in Supplement 2).

**Table 2.** Adjusted Differences in Outcome Measures^a^

Outcome	Mean (SD)			
	Control	Gamification		
		With support	With collaboration	With competition
**Physical activity, steps per day, No.**				
Baseline	4410 (2263)	4353 (2439)	4122 (2817)	4681 (2893)
Months 1-6	4202 (2185)	4854 (2539)	4445 (2669)	5066 (2882)
Difference relative to control (95% CI)	NA	691 (267 to 1115)	468 (43 to 892)	639 (204 to 1074)
*P* value	NA	.001	.03	.004
Months 1-12	4196 (2017)	4655 (2355)	4257 (2388)	5007 (2733)
Difference relative to control (95% CI)	NA	503 (103 to 903)	280 (−115 to 674)	606 (201 to 1011)
*P* value	NA	.01	.16	.003
**Weight, kg**				
Baseline	106.8 (22.5)	103.4 (18.2)	110.8 (21.4)	108.3 (20.7)
Month 6	104.4 (22.9)	99.9 (17.6)	108.1 (21.4)	106.6 (21.3)
Difference relative to control (95% CI)	NA	−1.4 (−3.0 to 0.3)	0.0 (−1.6 to 1.6)	0.7 (−1.0 to 2.4)
*P* value	NA	.11	1.00	.43
Month 12	105.1 (22.9)	99.7 (17.7)	107.5 (21.0)	105.4 (21.0)
Difference relative to control (95% CI)	NA	−1.9 (−4.2 to 0.3)	−1.5 (−3.8 to 0.9)	−1.1 (−3.5 to 1.3)
*P* value	NA	.09	.22	.36
**Hemoglobin A_1c_ level, %**				
Baseline	9.5 (1.5)	9.5 (1.5)	9.9 (1.7)	9.7 (1.6)
Month 6	8.8 (1.7)	9.1 (1.7)	8.7 (1.8)	8.8 (1.6)
Difference relative to control (95% CI)	NA	0.31 (−0.36 to 0.98)	−0.24 (−0.80 to 0.31)	−0.14 (−0.80 to 0.53)
*P* value	NA	.35	.38	.68
Month 12	8.8 (1.7)	8.9 (1.7)	8.8 (2.1)	8.7 (1.8)
Difference relative to control (95% CI)	NA	0.05 (−0.56 to 0.65)	−0.14 (−0.80 to 0.52)	−0.32 (−0.91 to 0.27)
*P* value	NA	.88	.68	.28
**LDL-C level, mg/dL**				
Baseline	99.9 (31.2)	101.7 (41.8)	97.9 (32.4)	90.0 (38.7)
Month 6	93.6 (33.0)	96.2 (36.1)	89.7 (32.2)	87.5 (34.6)
Difference relative to control (95% CI)	NA	1.1 (−10.5 to 12.7)	−0.6 (−10.9 to 9.7)	1.1 (−7.2 to 9.5)
*P* Value	NA	.85	.91	.79
Month 12	100.5 (31.8)	96.8 (31.0)	91.9 (28.5)	94.2 (32.9)
Difference relative to control (95% CI)	NA	−4.6 (−18.6 to 9.4)	−8.2 (−24.7 to 8.2)	−3.9 (−18.1 to 10.4)
*P* value	NA	.50	.29	.57

Abbreviations: LDL-C, low-density lipoprotein cholesterol; NA, not applicable.

SI conversion factors: To convert hemoglobin A_1c_ to proportion of total hemoglobin, multiply by 0.01; LDL-C to millimoles per liter, multiply by 0.0259.

^a^ Adjusted models used imputed data and adjusted for baseline outcome measure, calendar month, and study arm to report differences relative to controls.

In adjusted models examining the trajectory of physical activity from the first 6 months to the last 6 months of the trial, gamification with support decreased significantly (−423 steps; 95% CI, −624 to −223 steps; *P* = .003) but gamification with competition did not (−143 steps; 95% CI, −414 to 128 steps; *P* = .23). Gamification with collaboration decreased significantly (−394 steps; 95% CI, −512 to −276 steps; *P* < .001), but the control group did not change significantly (−43 steps; 95% CI, −235 to 149 steps; *P* = .60).

### Weight

The unadjusted mean weight by study arm at baseline, 6 months, and 12 months are depicted in Figure 2B. From baseline to 6 months, all study arms had decreases in mean weight. From month 6 to 12, the control arm had a slight increase in mean weight and the gamification arms had slight decreases. At 12 months, all study arms had significant decreases in mean weight from baseline as follows: −2.0 kg in the control arm (95% CI, −3.5 to −0.05 kg; *P* = .04), −3.6 kg in gamification with support (95% CI, −5.0 to −2.2 kg; *P* < .001), −3.4 kg in gamification with collaboration (95% CI, −4.7 to −2.0 kg; *P* < .001), and −2.9 kg in gamification with competition (95% CI, −4.8 to −1.1 kg; *P* < .001).

In the adjusted models, compared with the control group at 1 year, there were no significant changes in weight from baseline among participants in gamification with support (−1.9 kg; 95% CI, −4.2 to 0.3 kg; *P* = .09), gamification with collaboration (−1.5 kg; 95% CI, −3.8 to 0.9 kg; *P* = .22), or gamification with competition (−1.1 kg; 95% CI, −3.5 to 1.3 kg; *P* = .36) (Table 2). Results were similar in sensitivity analyses that used collected data without multiple imputation (eTable 3 in Supplement 2).

**Figure 3.**
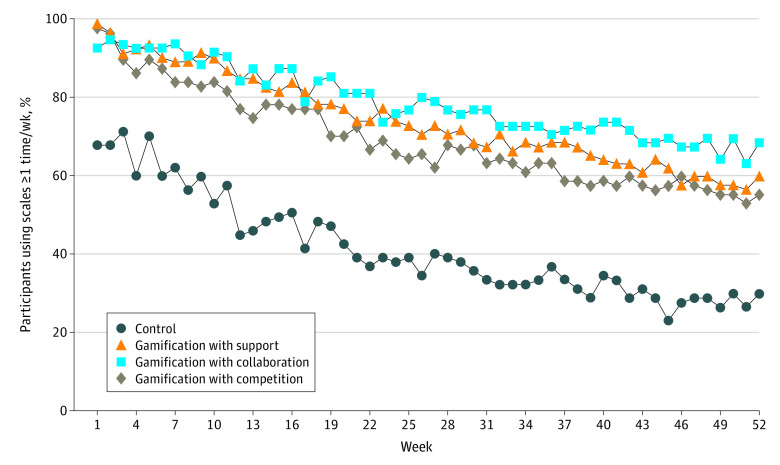
Smart Weight Scale Use Depicted are the proportion of participants who weighed in at least once per week by trial arm.

Figure 3 depicts the proportion of participants who weighed in at least once a week during the study period. All trial arms steadily decreased, but the gamification trial arms remained above 50% for 52 weeks while the control arm decreased to below 50% by week 12.

### HbA_1c_ and LDL-C Levels

Among all participants at baseline, 72.3% (261 of 361) were receiving insulin and 39.9% (144 of 361) were using an oral diabetes medication. The unadjusted mean HbA_1c_ levels by trial arm at baseline, 6 months, and 12 months are depicted in Figure 2C. The HbA_1c_ level declined in all arms by 6 months and then was mostly steady through 12 months. In the control arm, the unadjusted mean HbA_1c_ level was 9.5% at baseline, declined to 8.8% by 6 months, and was 8.8% by 12 months. In gamification with competition, the unadjusted mean HbA_1c_ level was 9.7% at baseline, declined to 8.8% by 6 months, and was 8.7% by 12 months.

In the adjusted models, compared with the control group at 1 year, there were no significant changes in HbA_1c_ levels from baseline among participants in any of the gamification arms (Table 2). Results were similar in sensitivity analyses that used collected data without multiple imputation (eTable 3 in Supplement 2).

Among all participants at baseline, 49.6% (179 of 361) reported using a statin medication. Baseline mean LDL-C levels were similar across trial arms and well controlled at baseline (Table 1). In the adjusted models, compared with the control group at 1 year, there were no significant changes in LDL-C levels from baseline among participants in any of the gamification arms (Table 2). Results were similar in sensitivity analyses that used collected data without multiple imputation (eTable 3 in Supplement 2).

### Safety and Patient Experience

During the trial, 1 person in the gamification with collaboration arm reported arthritic knee pain, which may have been related to increased physical activity. There were no other adverse events reported that were related to the trial. Three patients died from causes not related to the study.

At the end of the trial, most patients who responded to the survey reported satisfaction with the wearable device (80.2% [130 of 162]) and smart weight scale (85.8% [139 of 162]). Perceptions of the trial’s effect on outcomes were similar across arms, with overall rates of satisfaction at 71.6% (116 of 162) for increasing physical activity, 50.6% (82 of 162) for helping with weight loss, and 56.8% (92 of 162) for improving diabetes control (eTable 4 in Supplement 2).

## Discussion

In this trial of adults with overweight and obesity and uncontrolled type 2 diabetes, we found that gamification interventions with support or competition each significantly increased physical activity relative to the control group during the 1-year intervention. Increases in physical activity were sustained throughout 12 months among participants receiving gamification with competition but declined in the last 6 months among participants receiving gamification with support. Participants in all study arms had significant reductions in weight and HbA_1c_ levels from baseline, but none of the gamification interventions resulted in significant differences in these outcomes relative to the control group. To our knowledge, this is one of the only trials to test behaviorally designed gamification among patients with chronic disease over a 1-year period.

Physical activity increases relative to the control group among participants who received gamification with support (503 steps more per day) or competition (606 steps more per day) represented a 12% to 13% increase from baseline. This percentage increase from baseline is similar to prior gamification trials that were conducted among families in the Framingham Heart Cohort (13%) and employees with overweight or obesity from a large consulting firm (15%).^18,19^ Recent evidence indicates that even small increases or light-intensity physical activity can lead to health benefits, particularly for more sedentary individuals.^42,43,44,45^ LaCroix et al^45^ found that increases in light-intensity physical activity were associated with reductions in cardiovascular disease events. Other studies noted that increasing daily step counts was associated with lower rates of mortality in direct relationship until about 7500 steps per day.^46,47^ Future studies could evaluate ways to create adaptive goal setting or combine with other interventions to increase effect sizes and monitor health outcomes over longer periods.

Results from this and the 2 previous gamification trials^18,19^ revealed insights into the dynamic of social incentives that stakeholders should be taken into account when designing behavior change programs. Gamification with collaboration was effective in the trial with families from the Framingham Heart Cohort (support and competition were not tested in this study), but this trial was conducted in a setting in which members of each team knew each other well.^18^ Among the trials conducted with consulting employees who had uncontrolled type 2 diabetes, competition and support were effective, but only competition had sustained effects.^19^ Collaboration was not effective in either of these 2 trials. Collectively, these results may indicate that a collaborative social incentive works better when members of a group have strong preexisting social connections but otherwise may be relatively ineffective. In a clinical trial testing gamification with collaboration to promote weight loss, teams lost more weight if they lived together than if they lived separately (4.5 vs 2.7 kg),^20,29^ also supporting the notion that stronger social ties may be more responsive to collaborative social incentives. Gamification with support asks the participant to identify a family member or friend and notifies that person of progress, which may also leverage existing social connections. Gamification with competition may be better suited for interventions that involve individuals grouped together who have weaker social connections. Future studies could directly test the effects of social incentives based on differences in social connections within a group.

Although participants in 2 intervention arms had increases in physical activity, there were no significant changes relative to the control group in weight or HbA_1c_ levels. However, all trial arms had decreases in these measures from baseline. Participants in the control arm slightly gained weight in the last 6 months, but those in the intervention arms did not. Future studies could evaluate the longer-term effect on weight to identify whether gamification can help participants to maintain weight loss. The lack of improvement in these other outcomes could represent regression to the mean or that other factors may have played an important role. For example, we did not have information on participants’ diet or caloric intake. In addition, at baseline, 72.3% of the participants were receiving insulin and 39.9% were receiving an oral diabetes medication. Participation in the study and receipt of connected devices could have influenced medication adherence and be a reason that HbA_1c_ levels declined in all trial arms, including the control arm.

### Strengths and Limitations

This trial has several strengths. The sample was a high-risk population with uncontrolled type 2 diabetes, low baseline activity levels, and diversity in race/ethnicity, educational levels, and income. Gamification interventions incorporated scientific principles from behavioral economics. The program used an automated technology platform and was remotely monitored, which makes it more scalable than personnel-intensive approaches. The intervention lasted 1 year, which, to our knowledge, is the longest clinical trial conducted to date testing behaviorally designed gamification.

This trial has several limitations. First, participants were from a single health system and needed access to a smartphone or tablet, which may limit generalizability. However, more than 80% of US adults now own a smartphone.^48^ These interventions should be tested in other populations and settings. Second, we evaluated physical activity using step counts and did not have data on other measures of physical activity. Recent evidence and the current physical activity guidelines indicate that even small increases in light-intensity physical activity provide health benefits^42,43,44,45,49^; however, moderate to vigorous physical activity provides greater benefit and should be evaluated in future studies. Third, missing rates of step data varied by trial arm and were highest among the control group at about 40.5%. Fourth, we did not capture information on communications or interactions between the participants in the competition or collaboration groups and so are unable to further evaluate the success or lack thereof of these mechanisms in different groups. Fourth, although we adjusted our statistical threshold for multiple comparisons, we did not adjust for multiple primary outcomes. Fifth, this trial lasted 1 year, which, to our knowledge, is the longest of its kind. However, even longer-term evaluations are needed. Sixth, the interventions in this study were lighter touch digital approaches, and future studies may need to examine stronger ways to promote social incentives.

## Conclusions

In a remotely monitored randomized clinical trial of adults with overweight or obesity and uncontrolled type 2 diabetes, gamification interventions that encouraged social support or competition led to significant increases in physical activity relative to the control group during the 1-year intervention, but an intervention that encouraged collaboration among people who did not know each other did not. Gamification with competition had sustained increases of physical activity through 12 months, but activity levels in gamification with support declined in the last 6 months. All trial arms resulted in significant reductions in weight and HbA_1c_ levels from baseline, but none of the gamification interventions resulted in significant differences in these outcomes relative to the control group. The results of the iDiabetes trial indicate that gamification designed to incorporate behavioral insights and delivered through an automated and remotely monitored platform is a promising approach to increase physical activity among adults with type 2 diabetes but that gamification may need to be combined with other approaches to promote weight loss or changes in glycemic control.

## Notes

Supplement 1. Trial Protocol

Supplement 2. **eTable 1.** Missing Data Rates by Arm and Study Period **eTable 2.** Number of Observations Collected by Outcome and Time Period **eTable 3.** Adjusted Differences in Outcomes Without Multiple Imputation **eTable 4.** Patient Perceptions of Trial Experience From End of Study Surveys

Supplement 3. Data Sharing Statement

## References

[1] Centers for Disease Control and Prevention. National diabetes statistics report, 2020. Updated August 2020. Accessed October 1, 2020. https://www.cdc.gov/diabetes/data/statistics-report/index.html

[2] Centers for Disease Control and Prevention. Physical activity. Updated March 12, 2021. Accessed October 1, 2020. https://www.cdc.gov/physicalactivity/index.html

[3] Magkos F, Yannakoulia M, Chan JL, Mantzoros CS. Management of the metabolic syndrome and type 2 diabetes through lifestyle modification. Annu Rev Nutr. 2009;29:223-256. doi:10.1146/annurev-nutr-080508-141200 19400751 PMC5653262

[4] Chiu CJ, Wray LA. Factors predicting glycemic control in middle-aged and older adults with type 2 diabetes. Prev Chronic Dis. 2010;7(1):A08. 20040223 PMC2811503

[5] Campbell L, Rössner S. Management of obesity in patients with type 2 diabetes. Diabet Med. 2001;18(5):345-354. doi:10.1046/j.1464-5491.2001.00546.x 11472443

[6] Wing RR, Bolin P, Brancati FL, ; Look AHEAD Research Group. Cardiovascular effects of intensive lifestyle intervention in type 2 diabetes. N Engl J Med. 2013;369(2):145-154. doi:10.1056/NEJMoa1212914 23796131 PMC3791615

[7] Colberg SR, Sigal RJ, Yardley JE, . Physical activity/exercise and diabetes: a position statement of the American Diabetes Association. Diabetes Care. 2016;39(11):2065-2079. doi:10.2337/dc16-1728 27926890 PMC6908414

[8] Foreyt JP, Poston WS II. The challenge of diet, exercise and lifestyle modification in the management of the obese diabetic patient. Int J Obes Relat Metab Disord. 1999;23(suppl 7):S5-S11. doi:10.1038/sj.ijo.0800955 10455465

[9] Schroeder SA. Shattuck Lecture. We can do better—improving the health of the American people. N Engl J Med. 2007;357(12):1221-1228. doi:10.1056/NEJMsa073350 17881753

[10] Edwards EA, Lumsden J, Rivas C, . Gamification for health promotion: systematic review of behaviour change techniques in smartphone apps. BMJ Open. 2016;6(10):e012447. doi:10.1136/bmjopen-2016-012447 27707829 PMC5073629

[11] Kawachi I. It’s all in the game—the uses of gamification to motivate behavior change. JAMA Intern Med. 2017;177(11):1593-1594. doi:10.1001/jamainternmed.2017.4798 28973152

[12] Sardi L, Idri A, Fernández-Alemán JL. A systematic review of gamification in e-health. J Biomed Inform. 2017;71:31-48. doi:10.1016/j.jbi.2017.05.011 28536062

[13] Patel MS, Foschini L, Kurtzman GW, . Using wearable devices and smartphones to track physical activity: initial activation, sustained use, and step counts across sociodemographic characteristics in a national sample. Ann Intern Med. 2017;167(10):755-757. doi:10.7326/M17-1495 28973116

[14] Lenihan D. Health games: a key component for the evolution of wellness programs. Games Health J. 2012;1(3):233-235. doi:10.1089/g4h.2012.0022 26193441

[15] Lowensteyn I, Berberian V, Berger C, Da Costa D, Joseph L, Grover SA. The sustainability of a workplace wellness program that incorporates gamification principles: participant engagement and health benefits after 2 years. Am J Health Promot. 2019;33(6):850-858. doi:10.1177/0890117118823165 30665309

[16] Cotton V, Patel MS. Gamification use and design in popular health and fitness mobile applications. Am J Health Promot. 2019;33(3):448-451. doi:10.1177/0890117118790394 30049225 PMC6348030

[17] Hamari J KJ, Sarsa H. Does gamification work? a literature review of empirical studies on gamification. Paper presented at: 47th Hawaii International Conference on System Sciences; January 6-9, 2014; Waikoloa, Hawaii.

[18] Patel MS, Benjamin EJ, Volpp KG, . Effect of a game-based intervention designed to enhance social incentives to increase physical activity among families: the BE FIT randomized clinical trial. JAMA Intern Med. 2017;177(11):1586-1593. doi:10.1001/jamainternmed.2017.3458 28973115 PMC5710273

[19] Patel MS, Small DS, Harrison JD, . Effectiveness of behaviorally designed gamification interventions with social incentives for increasing physical activity among overweight and obese adults across the United States: the STEP UP randomized clinical trial. JAMA Intern Med. 2019;179(12):1624-1632. doi:10.1001/jamainternmed.2019.3505 31498375 PMC6735420

[20] Kurtzman GW, Day SC, Small DS, . Social incentives and gamification to promote weight loss: the LOSE IT randomized, controlled trial. J Gen Intern Med. 2018;33(10):1669-1675. doi:10.1007/s11606-018-4552-1 30003481 PMC6153249

[21] Asch DA, Volpp KG. On the Way to Health. LDI Issue Brief. 2012;17(9):1-4. 22934330

[22] Fortunato M, Harrison J, Oon AL, . Remotely monitored gamification and social incentives to improve glycemic control among adults with uncontrolled type 2 diabetes (iDiabetes): protocol for a randomized controlled trial. JMIR Res Protoc. 2019;8(11):e14180. doi:10.2196/14180 31746765 PMC6893558

[23] Chokshi NP, Adusumalli S, Small DS, . Loss-framed financial incentives and personalized goal-setting to increase physical activity among ischemic heart disease patients using wearable devices: the ACTIVE REWARD randomized trial. J Am Heart Assoc. 2018;7(12):e009173. doi:10.1161/JAHA.118.009173 29899015 PMC6220554

[24] Patel MS, Asch DA, Rosin R, . Individual versus team-based financial incentives to increase physical activity: a randomized, controlled trial. J Gen Intern Med. 2016;31(7):746-754. doi:10.1007/s11606-016-3627-0 26976287 PMC4907949

[25] Patel MS, Asch DA, Rosin R, . Framing financial incentives to increase physical activity among overweight and obese adults: a randomized, controlled trial. Ann Intern Med. 2016;164(6):385-394. doi:10.7326/M15-1635 26881417 PMC6029433

[26] Patel MS, Volpp KG, Rosin R, . A randomized trial of social comparison feedback and financial incentives to increase physical activity. Am J Health Promot. 2016;30(6):416-424. doi:10.1177/0890117116658195 27422252 PMC6029434

[27] Patel MS, Volpp KG, Rosin R, . A randomized, controlled trial of lottery-based financial incentives to increase physical activity among overweight and obese adults. Am J Health Promot. 2018;32(7):1568-1575. doi:10.1177/0890117118758932 29534597

[28] Case MA, Burwick HA, Volpp KG, Patel MS. Accuracy of smartphone applications and wearable devices for tracking physical activity data. JAMA. 2015;313(6):625-626. doi:10.1001/jama.2014.17841 25668268

[29] Lienert J, Patel M. Patient phenotypes help explain variation in response to a social gamification weight loss intervention. Am J Health Promot. 2020;34(3):277-284. doi:10.1177/0890117119892776 31876175

[30] Centers for Disease Control and Prevention. Diabetes. Updated March 30, 2021. Accessed October 1, 2020. https://www.cdc.gov/diabetes/index.html

[31] Ariely D, Wertenbroch K. Procrastination, deadlines, and performance: self-control by precommitment. Psychol Sci. 2002;13(3):219-224. doi:10.1111/1467-9280.00441 12009041

[32] Rogers T, Milkman KL, Volpp KG. Commitment devices: using initiatives to change behavior. JAMA. 2014;311(20):2065-2066. doi:10.1001/jama.2014.3485 24777472

[33] Kahneman D, Tversky A. Prospect theory: an analysis of decision under risk. Econometrica. 1979;47(2):263-292. doi:10.2307/1914185

[34] Dai H, Milkman KL, Riis J. The fresh start effect: temporal landmarks motivate aspirational behavior. Manage Sci. 2014;60(10):2563-2582. doi:10.1287/mnsc.2014.1901

[35] Asch DA, Rosin R. Engineering social incentives for health. N Engl J Med. 2016;375(26):2511-2513. doi:10.1056/NEJMp1603978 28029924

[36] Sen AP, Sewell TB, Riley EB, . Financial incentives for home-based health monitoring: a randomized controlled trial. J Gen Intern Med. 2014;29(5):770-777. doi:10.1007/s11606-014-2778-0 24522623 PMC4000326

[37] Long JA, Jahnle EC, Richardson DM, Loewenstein G, Volpp KG. Peer mentoring and financial incentives to improve glucose control in African American veterans: a randomized trial. Ann Intern Med. 2012;156(6):416-424. doi:10.7326/0003-4819-156-6-201203200-00004 22431674 PMC3475415

[38] Asch DA, Troxel AB, Stewart WF, . Effect of financial incentives to physicians, patients, or both on lipid levels: a randomized clinical trial. JAMA. 2015;314(18):1926-1935. doi:10.1001/jama.2015.14850 26547464 PMC5509443

[39] Young R, Johnson DR. Handling missing values in longitudinal panel data with multiple imputation. J Marriage Fam. 2015;77(1):277-294. doi:10.1111/jomf.12144 26113748 PMC4477955

[40] Prochaska JO, Velicer WF. The transtheoretical model of health behavior change. Am J Health Promot. 1997;12(1):38-48. doi:10.4278/0890-1171-12.1.38 10170434

[41] Rubin DB. Multiple Imputation for Nonresponse in Surveys. Wiley; 1987.

[42] Piercy KL, Troiano RP, Ballard RM, . The physical activity guidelines for Americans. JAMA. 2018;320(19):2020-2028. doi:10.1001/jama.2018.14854 30418471 PMC9582631

[43] Thompson PD, Eijsvogels TMH. New physical activity guidelines: a call to activity for clinicians and patients. JAMA. 2018;320(19):1983-1984. doi:10.1001/jama.2018.16070 30418469

[44] Giroir BP, Wright D. Physical activity guidelines for health and prosperity in the United States. JAMA. 2018;320(19):1971-1972. doi:10.1001/jama.2018.16998 30418473

[45] LaCroix AZ, Bellettiere J, Rillamas-Sun E, ; Women’s Health Initiative (WHI). Association of light physical activity measured by accelerometry and incidence of coronary heart disease and cardiovascular disease in older women. JAMA Netw Open. 2019;2(3):e190419. doi:10.1001/jamanetworkopen.2019.0419 30874775 PMC6484645

[46] Lee IM, Shiroma EJ, Kamada M, Bassett DR, Matthews CE, Buring JE. Association of step volume and intensity with all-cause mortality in older women. JAMA Intern Med. 2019;179(8):1105-1112. doi:10.1001/jamainternmed.2019.0899 31141585 PMC6547157

[47] Saint-Maurice PF, Troiano RP, Bassett DR Jr, . Association of daily step count and step intensity with mortality among US adults. JAMA. 2020;323(12):1151-1160. doi:10.1001/jama.2020.1382 32207799 PMC7093766

[48] Pew Research Center. Mobile fact sheet. Updated April 7, 2021. Accessed September 30, 2020. https://www.pewinternet.org/fact-sheet/mobile/

[49] Spartano NL, Davis-Plourde KL, Himali JJ, . Association of accelerometer-measured light-intensity physical activity with brain volume: the Framingham Heart Study. JAMA Netw Open. 2019;2(4):e192745. doi:10.1001/jamanetworkopen.2019.2745 31002329 PMC6481600

