# Supplementary material for: Effect of Behaviorally Designed Gamification With Social Incentives on Lifestyle Modification Among Adults With Uncontrolled Diabetes: A Randomized Clinical Trial
Source: JAMA Netw Open. 2021 May 24;4(5):e2110255. doi: 10.1001/jamanetworkopen.2021.10255 (PMC8144928; doi:10.1001/jamanetworkopen.2021.10255)
Supplement: Supplement 1. — Trial Protocol [file jamanetwopen-e2110255-s001.pdf]

Supplement 1

Study Protocol

Table of Contents

1. Summary of changes to the protocol

2. Original Study Protocol

3. Final Study Protocol

Summary of Changes to the Protocol

Two changes were made to the protocol to expand recruitment to a broader sample of type 2 diabetics. First, we reduced the minimum body mass index requirement from 30 to 25 to allow patients who were overweight but not obese to enroll in the study. Second, some patients had very low baseline daily step count levels. In prior work, we did not count daily step values of less than 1000 steps per day during baseline assessment or in the study because they were though to reflect days on which the wearable was not worn for the full day. However, in this sample participant baseline physical activity levels were much lower than past trials. So, we allowed participants with baseline step counts less than 1000 steps per day to enroll. Therefore, we changed our main analysis to match the baseline assessment and include all recorded step values.

Original Study Protocol

A Randomized, Controlled Trial Using Social Incentives and Gamification to Change Health Behaviors and Improve Glycemic Control Among Type 2 Diabetics

Study Protocol

October 31, 2016

Outline

1. Abstract

2. Overall objectives

3. Aims

 3.1 Primary outcome

 3.2 Secondary outcomes

4. Background

5. Study design

 5.1 Design

 5.2 Study duration

 5.3 Target population

 5.4 Accrual

 5.5 Key inclusion criteria

 5.6 Key exclusion criteria

6. Subject recruitment

7. Subject compensation

8. Study procedures

 8.1 Consent

 8.2 Procedures

9. Analysis plan

10. Investigators

11. Human research protection

 11.1 Data confidentiality

 11.2 Subject confidentiality

 11.3 Subject privacy

11.4 Data disclosure

11.5 Data safety and monitoring

11.6 Risk/benefit

11.6.1 Potential study risks

11.6.2 Potential study benefits

11.6.3 Risk/benefit assessment

1. Abstract

Type 2 diabetes is a significant cause of morbidity and mortality in the United States. There is strong evidence that lifestyle modification including increasing physical activity and losing weight are effective for improving glycemic control. Insights from behavioral economics have shown promise for motivating behavior change through the use of financial incentives. However, social incentives or those influences that impact individuals to adjust their inherent behaviors based on social ties and connections have not been well examined. Social incentives are a more patient-centered approach that leverages and enhances the existing connections and influences on the individual. Since one individual's behavior change is connected to many others within their network, social incentives have the potential to be a scalable intervention that impacts population health. Gamification, or the use of game design in non-game situations, is often used in the real world, but social incentives are not often incorporated in the approach. In this study, we will conduct a one-year, four-arm, randomized, controlled trial to compare three social incentive-based gamification interventions to control. Each intervention will use insights from behavioral economics to leverage social incentives and gamification.

2. Overall objectives

The objective of this study is to use a randomized, controlled trial to test the effectiveness of three interventions using social incentives and gamification to promote physical activity, weight loss, and improved glycemic control among adult type 2 diabetics.

3. Aims

3.1 Primary outcome

The primary outcome variables are change in mean daily steps, weight in pounds, and hemoglobin A1c from baseline to end of the one-year study.

3.2 Secondary outcome

Secondary outcomes include the change in LDL-C levels from baseline to end of the one-year study period, as well as change in mean daily steps, weight in pounds, hemoglobin A1c, and LDL-C from baseline to the six-month midpoint of the study period.

4. Background

Type 2 diabetes is a significant cause of morbidity and mortality in the United States (1-2). There is strong evidence that lifestyle modification including increasing physical activity and losing weight are effective for improving glycemic control (3-4). However, there are several important reasons why these interventions are challenging to accomplish at broader scale. First, they involve behaviors that occur mostly outside of clinical encounters and instead within the everyday lives of patients (5). Second, past interventions targeting these behaviors have been

more personnel-intensive and therefore they are costly and complex to deploy at larger scale (5). Third, most interventions are based on the premise that individuals behave rationally and that if they are educated on the reasons for improving their health, that they will change their behaviors accordingly. However, we know that individuals do not behave in this manner (6) For example, many people know that physical inactivity and obesity is not good for their health, yet they don't regularly exercise or change their diet for weight loss. In the United States, more than 50% of adults do not obtain enough physical activity and more than 70% of adults are overweight or obese (7-8). Therefore, there is a significant need for new interventions that can passively collect data on daily health behaviors and combine automated feedback with behavioral strategies that anticipate and leverage predictable barriers to behavior change.

Behavioral economics is a field of study that incorporates principles from psychology to help us understand why individuals make decisions that are not aligned with their longer-term health goals. Instead, people often deviate from these goals in a predictable manner and from a common set of decision errors (6). For example, people tend to be more motivated by immediate rather than delayed gratification (9), by losses rather than gains (10), and they tend to take actions to avoid the feeling of regret (11). These insights have been incorporated in financial incentive-based approaches designed by our research group and resulted in improvements in weight loss (12-14), physical activity (15-17), smoking cessation (18-19), and adherence to remote-monitoring devices that collect patient-generated health data (20).

While financial incentives designed using behavioral economics have demonstrated success in motivating behavior change, social incentives or those influences that impact individuals to adjust their behaviors based on social ties and connections have not been well examined. Since each individual's behavior change is connected to many others within their network (21-22), social incentives have the potential to be a scalable intervention that impacts population health. There is evidence suggesting that social incentives can be very powerful in influencing behavior. For example, in a large retrospective study an individual's chances of becoming obese was 57% higher if a close friend became obese, 40% higher if a sibling became obese, and 37% higher if a spouse became obese (21). In comparison, when an individual's spouse or partner loses weight, the individual is three times more likely to also lose weight (23). Therefore, there is a significant opportunity to improve health by better understanding how to design social incentive interventions to motivate behavior change.

[1] Gregg EW, Li Y, Wang J, Burrows NR, Ali MK, Rolka D, Williams DE, Geiss L. Changes in diabetes-related complications in the United States, 1990-2010. N Engl J Med. 2014;370(16):1514-23. [2] Menke A, Casagrande S, Geiss L, Cowie CC. Prevalence of and Trends in Diabetes Among Adults in the United States, 1988-2012. JAMA. 2015;314(10):1021-9. [3] Pronk NP, Remington PL. Combined Diet and Physical Activity Promotion Programs for Prevention of Diabetes: Community Preventive Services Task Force Recommendation Statement. Ann Intern Med. 2015;163(6):465-469. [4] Jeon CY, Lokken RP, Hu FB, van Dam RM. Physical activity of moderate intensity and risk of type 2 diabetes: a systematic review.

Diabetes Care. 2007;30(3):744-52. [5] Asch DA, Muller RW, Volpp KG. Automated hovering in health care--watching over the 5000 hours. N Engl J Med. 2012;367(1):1-3. [6] Loewenstein G, Brennan T, Volpp KG. Asymmetric paternalism to improve health behaviors. JAMA. 2007;298(20):2415-7. [7] Centers for Disease Control and Prevention. Facts about physical activity. Accessed at www.cdc.gov/physicalactivity/data/facts.html [8] Ogden CL, Carroll MD, Kit BK, Flegal KM. Prevalence of childhood and adult obesity in the United States, 2011–2012. JAMA. 2014;311(8): 806–14. [9] O'Donoghue T, Rabin M. The economics of immediate gratification. Journal of Behavioral Decision Making. 2000;13:233-250. [10] Kahneman D, Tversky A. Prospect theory: an analysis of decision under risk. Econometrica. 1979;47:263-91. [11] Zeelenberg M, Pieters R. Consequences of regret aversion in real life: the case of the Dutch postcode lottery. Organ Behav Hum Decis Process. 2004;93:155-68. [12] Volpp KG, John LK, Troxel AB, Norton L, Fassbender J, Loewenstein G. Financial incentive-based approaches for weight loss: a randomized trial. JAMA. 2008;300(22):2631-7. [13] John LK, Loewenstein G, Troxel AB, Norton L, Fassbender JE, Volpp KG. Financial incentives for extended weight loss: a randomized, controlled trial. J Gen Intern Med. 2011:26(6):621–626. [14] Kullgren JT, Troxel AB, Loewenstein G, Asch DA, Norton LA, Wesby L, Tao Y, Zhu J, Volpp KG. Individual- versus group-based financial incentives for weight loss: a randomized, controlled trial. Ann Intern Med. 2013;158(7):505-14. [15] Patel MS, Asch DA, Rosin R, Small DS, Bellamy SL, Heuer J, Sproat S, Hyson C, Haff N, Lee SM, Wesby L, Hoffer K, Shuttleworth D, Taylor D, Hilbert V, Zhu J, Yang L, Wang X, Volpp KG. Framing financial incentives to increase physical activity among overweight and obese adults: a randomized, controlled trial. Ann Intern Med. 2016;164(6):385-396. [16] Patel MS, Asch DA, Rosin R, Small DS, Bellamy SL, Eberbach K, Walters KJ, Haff N, Lee SM, Wesby L, Hoffer K, Shuttleworth D, Taylor D, Hilbert V, Zhu J, Yang L, Wang X, Volpp KG. Individual versus team-based financial incentives to increase physical activity: a randomized, controlled trial. J Gen Intern Med. . 2016;Published Online March 14, 2016. DOI: 10.1007/s11606-016-3627-0. [17] Patel MS, Volpp KG, Rosin R, Bellamy SL, Small DS, Fletcher MA, Osman-Koss R, Brady JL, Haff N, Lee SM, Wesby L, Hoffer K, Shuttleworth D, Taylor DH, Ulrich V, Zhu J, Yang L, Wang X, Asch DA. A Randomized Trial of Social Comparison Feedback and Financial Incentives to Increase Physical Activity. Am J Health Promot. In Press. [18] Volpp KG, Troxel AB, Pauly MV, Glick HA, Puig A, Asch DA, Galvin R, Zhu J, Wan F, DeGuzman J, Corbett E, Weiner J, Audrain-McGovern J. A randomized, controlled trial of financial incentives for smoking cessation. N Engl J Med. 2009;360(7):699-709. [19] Halpern SD, French B, Small DS, Saulsgiver K, Harhay MO, Audrain-McGovern J, Loewenstein G, Brennan TA, Asch DA, Volpp KG. Randomized trial of four financial-incentive programs for smoking cessation. N Engl J Med. 2015;372(22):2108-17. [20] Sen AP, Sewell TB, Riley EB, Stearman B, Bellamy SL, Hu MF, Tao Y, Zhu J, Park JD, Loewenstein G, Asch DA, Volpp KG. Financial incentives for home-based health monitoring: a randomized controlled trial. J Gen Intern Med. 2014;29(5):770-7. [21] Christakis NA, Fowler JH. The spread of obesity in a large social network over 32 years. N Engl J Med. 2007;357(4):370-9. [22] Christakis NA, Fowler JH. The collective dynamics of smoking in a

large social network. N Engl J Med. 2008;358(21):2249-58. [23] Jackson SE, Steptoe A, Wardle J. The Influence of Partner's Behavior on Health Behavior Change: The English Longitudinal Study of Ageing. JAMA Intern Med. 2015;175(3):385-92.

5. Study design

5.1 Design

This is a four-arm, one-year randomized, controlled trial. The study will be conducted using Way to Health, an automated information technology platform at the University of Pennsylvania that integrates wireless devices, conducts clinical trial randomization and enrollment processes, delivers messaging (text or email) delivers self-administered surveys, automates payment transfers, and securely captures data for research purposes.

The study team will identify potential participants from the electronic health record at the University of Pennsylvania Health System using Penn Data Store and Clarity, an EPIC reporting database. Patients with a hemoglobin A1c of 8.0 or greater and body mass index of 30 or greater will receive either an email, letter, phone call, or some combination from the study team introducing the study and its eligibility criteria with instructions on how to learn more and begin the enrollment process. Interested participants will be instructed to visit the study website to create an account, review and complete informed consent, and complete eligibility survey. Eligible participants will be instructed to obtain a hemoglobin A1c and LDL-C laboratory test. If the hemoglobin A1c is 8.0 or greater and the participant is still interested in continuing the enrollment process, a wearable activity tracking device will be mailed to them to wear for about two weeks to collect a baseline step count. After that, the participant will be scheduled for an in-person visit with the study team to complete the enrollment process including surveys. During this process the participant will be told that they may be randomized to participate individually or on a team with up to two other members. If they have a family member, friend, or coworker that might be interested in also participating, they can let the study team know that they would like to be randomized together.

During the in-person visit, all participants will receive education on the importance of diet and physical activity for weight loss and glycemic control. Each participant will have already received a wearable activity tracker and will be informed that he or she should wear this device during the one-year study period. Each participant will be giving a wireless weight scale for monitoring their weight at home. They will establish a baseline weight measure at the in-person visit. Since weight measures may vary based on time of day and in relation to last meal, we will monitor their first weight at home and if 5 lbs. or greater different will call the patient to assess which weight is closer to their true weight and use that as their baseline. In addition, while we are targeting patients with body mass index of 30 or greater, we will allow those that have body mass index of 28 or greater at the in-person visit to avoid not enrolling a participant due to regular fluctuations in weight.

Participants will be randomly assigned in blocks of four groups to one of the four study arms stratified based on whether the group of individuals already knew each other or not. Groups will have three individuals unless the individuals were already socially connected prior to the study, in which case they will be allowed to be randomized as a group of two or three. Participants randomized to the control arm will receive no other interventions.

--Goal Setting--

Each participant in the intervention arms will be asked to choose their goals for the study as follows: 1) Choose a weight loss goal that is 6%, 7%, or 8% of their baseline weight rounded up to the next pound; 2) Choose a daily step count goal that is 33%, 40%, or 50% greater than their baseline rounded up to the nearest hundred, or choose their own step goal as long as it is at least 1500 steps above their baseline; 3) Choose a HbA1c reduction goal of 1.5%, 2%, or 2.5%.

The weight loss goal will be set for a gradual decline over 26 weeks. The participant is given a weight target for each week in the study and if not achieved, then the target remains the same for the following week.

Participants will have a 4-week ramp up towards their step goal. The net difference between baseline and their goal will be divided by 4 and the participant will be asked to achieve the 25% increase each week for 4-week and then maintain the step goal for the remaining study period. For example, a participant with a baseline of 6000 steps and goal of 8000 steps will be asked to achieve goals of 6500, 7000, 7500, and 8000 for each of the first four weeks of the study. The they will be asked to maintain the 8000 step goal.

The HbA1c goal will be set for the 6-month mark and be expected to be maintained through the one-year mark.

At 3, 6, and 9 months, participants struggling to meet their goals (defined as in the blue or bronze levels of the game, see below) will be called to inquire about their progress in the study, moved up to the silver level, and given the opportunity to readjust their goals among the choices above.

All participants will receive $50 to enroll in the study (defined as being randomized and starting the intervention), $50 to obtain lab tests (HbA1c and LDL-C) and conduct a virtual weigh-in at home by facetime or skype after 6 months, $50 to obtain lab tests (HbA1c and LDL-C) and conduct a virtual weigh-in at home by facetime or skype after one year.

--Social Incentive Interventions (Arms 2-4)--

Participants in arms 2-4 will be entered into an intervention approach that has points and levels designed to incorporate insights from behavioral economics. On a daily basis, they will be asked to weigh-in. They will have a weekly weight target and mean step goal. First, participants will be asked to sign a pre-commitment pledge to strive to achieve their goals during the study. Pre-commitment has been demonstrated to motivate behavior change. Second, at the beginning of

each week the participant receives 70 points (10 for each day that week). If the participant does not weigh-in, they lose 10 points from their balance. This leverages loss aversion, which has been demonstrated to motivate behavior change more effectively with losses than gains. Third, at the end of each week if you have at least 40 points, met your weekly weight and step goal, you will move up a level (levels from lowest to highest: blue, bronze, silver, gold, platinum). If not, participants will drop a level. All participants begin at the silver level. Each week, participants get a fresh set of 70 points on Monday.

Weekly feedback will differ among the three arms to induce the different social incentives (support, competition, and collaboration) as follows:

Arm 2 – Supportive social incentive intervention: Participants in this arm will be asked to identify a family member or friend to be their support sponsor. A weekly report will be sent to this person with the participant's performance (goal achievement, points and level). A monthly report will be sent to the participant's primary care physician (PCP) with data on their change in weight, step goals, HbA1c, and LDL-C. A copy of this letter will be sent to the participant. The sponsor and PCP will serve as part of the participant's support network and be encouraged to reach out to the participant.

Arm 3 – Competitive social incentive intervention: Participants in this arm will be in a group of two or three people. At the end of each week the participants will receive an email with leaderboards. The first will rank the group members by points for that week. The second will rank the four members for the entire study thus far, displaying their total points and current level. This feedback may help to induce participants to compete for the top spot among the group. A monthly report will be sent to the participant's primary care physician (PCP) with data on their change in weight, step goals, HbA1c, and LDL-C. A copy of this letter will be sent to the participant.

Arm 4 – Collaborative social incentive intervention: Participants in this arm will be in a group of two or three people. Each day one of members of the group will be randomly selected to represent their team for that day. If they met their goal on the previous day (e.g. weighed in), everyone on the team keeps their points. If they didn't meet their goal then everyone on the team loses 10 points. In this design, each person is accountable to the others on the team and this may induce a collaborative effort to meet their daily goals. The entire team moves up a level only if all team members met their weekly weight and step targets. A monthly report will be sent to the participant's primary care physician (PCP) with data on their change in weight, step goals, HbA1c, and LDL-C. A copy of this letter will be sent to the participant.

Participants will have the opportunity to win a trophy, plaque, or medal depending on their level at the end of the study.

5.2 Study duration

This is one-year study.

5.3 Target population

Adults type 2 diabetics age 18 years to 70 years with a body mass index of 30 or greater and a HbA1c of 8 or greater.

5.4 Accrual

We estimate that a sample size of 360 participants (90 per arm) will provide at least 80% power using a conservative Bonferroni adjustment of the Type I error rate with a 2-sided α of 0.017 and accounting for a dropout rate of 10% to detect: 1) a 1100 step change in physical activity with a standard deviation of 2000 steps; 2) a 6 lb. change in weight with a standard deviation of 10 lbs; and 3) a 0.8% change in HbA1c with a standard deviation of 1.5%.

5.5 Key inclusion criteria

1) Age 18 years to 70 years; 2) ability to read and provide informed consent to participate in the study; 3) diagnosis of type 2 diabetes with a hemoglobin A1c of 8.0 or greater; 4) Self-reported body mass index (BMI) of 30 or greater. However, since the self-report may not be accurate and because weight can fluctuate, when they arrive for the in-person baseline weigh-in they will be allowed to participate if their BMI is 28 or greater; 5) Smartphone or tablet compatible with the application for the wearable activity tracking device and wireless weight scale.

5.6 Key exclusion criteria

1) Conditions that would make participation infeasible such as inability to provide informed consent, illiteracy or inability to speak, read, and write English; 2) conditions that would make participation unsafe such as pregnancy, previous diagnosis of an eating disorder, or history of unsafe weight loss practices; 3) already enrolled in another study targeting physical activity, weight loss, or glycemic control; 4) any other medical conditions or reasons he or she is unable to participate in the study for one year.

6. Subject recruitment

The study team will identify potential participants from the electronic health record at the University of Pennsylvania Health System using Penn Data Store and Clarity, an EPIC reporting database. Patients with a hemoglobin A1c of 8.0 or greater and body mass index of 30 or greater will receive either an email, letter, phone call, or some combination from the study team introducing the study and its eligibility criteria with instructions on how to learn more and begin the enrollment process. We may also post flyers in UPHS primary care facilities and endocrinologist clinics.

7. Subject compensation

All participants will receive $50 to enroll in the study (defined as being randomized and starting the intervention), $50 to obtain lab tests (HbA1c and LDL-C) and conduct a virtual weigh-in at home by facetime or skype after 6 months, $50 to obtain lab tests (HbA1c and LDL-C) and conduct a virtual weigh-in at home by facetime or skype after one year.

8. Study procedures

8.1 Consent

Upon recruitment, individuals who are interested in learning more about the study will be directed to the Way to Health web portal. Upon reaching the portal, potential participants will be asked to create an account and will then be informed of the details of the study, including its objectives, duration, requirements, and financial payments. If participants are still interested in participating, the Way to Health portal will take them through an automated online informed consent. The consent document will be divided into sections and potential participants will have to click a button to advance through each section. This is to help ensure that participants read the consent form thoroughly by breaking down the form into manageable blocks of text. Each section will have a button allowing the user to contact a researcher via email or by telephone if they have questions about the consent form. Successive screens will explain the voluntary nature of the study, the risks and benefits of participation, alternatives to participation, and that participants can withdraw from the study at any time. On the final consent screen, potential participants who click a clearly delineated button stating that they agree to participate in the study will be considered to have consented to enroll. Participants will be provided with details regarding how to contact the research team via email or phone at any time if they subsequently wish to withdraw from the study. This contact information will remain easily accessible via the participants' individual Way to Health web portal dashboards throughout the study. When the participant conducts the in-person meeting with the study team, they will be asked to physically sign a copy of the informed consent.

8.2 Procedures

After providing informed consent, participants will complete an online questionnaire to determine their eligibility and complete the study surveys. Eligible participants will be instructed to obtain a hemoglobin A1c and LDL-C laboratory test. These laboratory test measure the body's glucose and cholesterol control and will require no more than two vials of blood drawn. During the study, participants will be asked to obtain these measures at baseline and after 6 and 12 months. If the hemoglobin A1c is 8.0 or greater at baseline and the participant is still interested in continuing the enrollment process, a wearable activity tracking device will be mailed to them to wear for about two weeks to collect a baseline step count. After that, the participant will be scheduled for an in-person visit with the study team to complete the enrollment process. During this process the participant will be told that they may be randomized to participate individually or on a team with up to two other members. If they have a family

member, friend, or coworker that might be interested in also participating, they can let the study team know that they would like to be randomized together.

During the in-person visit, all participants will receive education on the importance of diet and physical activity for weight loss and glycemic control. Each participant will have already received a wearable activity tracker and will be informed that he or she should wear this device all the time (during the day and while sleeping) during the one-year study period. Each participant will be giving a wireless weight scale for monitoring their weight at home. They will establish a baseline weight measure at the in-person visit. Since weight measures may vary based on time of day and in relation to last meal, we will monitor their first weight at home and if more than 5 lbs. different will call the patient to assess which weight is closer to their true weight and use that as their baseline. In addition, while we are targeting patients with body mass index of 30 or greater, we will allow those that have body mass index of 28 or greater at the in-person visit to avoid not enrolling a participant due to regular fluctuations in weight.

Participants will be randomly assigned in blocks of four groups to one of the four study arms stratified based on whether the group of individuals already knew each other or not. Groups will have three individuals unless the individuals were already socially connected prior to the study, in which case they will be allowed to be randomized as a group of two or three. Participants randomized to the control arm will receive no other interventions.

9. Analysis plan

To compare sample characteristics between arms we will use t-tests or Wilcoxon rank-sum tests (F-tests or Kruskal-Wallis test) for continuous variables and Pearson chi square tests or Fisher's exact tests for categorical variables. In our primary analyses, we will compare change in mean daily steps (baseline vs. one-year study period), weight in pounds (baseline vs. end of one-year study period), and hemoglobin A1c (baseline vs. end of one-year study period) for each intervention arm compared to the control arm. In the secondary analysis we will compare change in LDL-C from baseline to the end of one-year study period. For each of the four measures listed we will also compare outcomes after 6-months. We will compare proportion of time the step goals were achieved during the one-year study. Our primary analyses will use multiple imputation for step data that are missing or have values less than 1000 steps since evidence suggests these values are likely not to be accurate measures of physical activity, for missing weight measures or missing hemoglobin A1c measures. We will conduct a series of adjusted regression models that include controlling for baseline measures, calendar month fixed effects, repeated measures and other participant characteristics such as demographics, comorbidities, self-reported measures, and validated survey measures. All hypothesis tests will be two-sided using a two-sided alpha of 0.017 as our threshold for statistical significance. We will use Stata and/or SAS to analyze the data.

We will conduct exploratory analyses on differences in sleep measures such as duration and quality and differences in heart rate measures such as baseline, average, and peak. We will conduct an exploratory qualitative evaluation of the survey free responses.

10. Investigators

Mitesh Patel, MD, MBA, MS is the Principal Investigator (PI) and is an Assistant Professor of Medicine and Health Care Management at the Perelman School of Medicine and The Wharton School at the University of Pennsylvania. He has past experience leading randomized, clinical trials using the Way to Health Platform to deploy interventions using financial and social incentives to promote weight loss and increased physical activity. He currently spends 80% of his effort on research and 20% on clinical and teaching activities.

11. Human research protection

11.1 Data confidentiality

Paper-based records will be kept in a secure location and only be accessible to personnel involved in the study. Computer-based files will only be made available to personnel involved in the study through the use of access privileges and passwords. Wherever feasible, identifiers will be removed from study-related information. Precautions are in place to ensure the data are secure by using passwords and encryption, because the research involves web-based surveys.

11.2 Subject confidentiality

Research material will be obtained from participant surveys, laboratory testing, wearable devices, and weight scales. All participants will provide informed consent for access to these materials. The data to be collected include demographic data (e.g., age, sex, self-identified race), hemoglobin A1c, LDL-C, step, sleep, and weight data. Research material that is obtained will be used for research purposes only. The same procedure used for the analysis of automated data sources to ensure protection of patient information will be used for the survey data, in that patient identifiers will be used only for linkage purposes or to contact patients. The study identification number, and not other identifying information, will be used on all data collection instruments. All study staff will be reminded to appreciate the confidential nature of the data collected and contained in these databases. The Penn Medicine Academic Computing Services (PMACS) will be the hub for the hardware and database infrastructure that will support the project and is where the Way to Health web portal is based. The PMACS is a joint effort of the University of Pennsylvania's Abramson Cancer Center, the Cardiovascular Institute, the Department of Pathology, and the Leonard Davis Institute. The PMACS provides a secure computing environment for a large volume of highly sensitive data, including clinical, genetic, socioeconomic, and financial information. Among the IT projects currently managed by PMACS are: (1) the capture and organization of complex, longitudinal clinical data via web and clinical applications portals from cancer patients enrolled in clinical trials; (2) the integration of genetic

array databases and clinical data obtained from patients with cardiovascular disease; (3) computational biology and cytometry database management and analyses; (4) economic and health policy research using Medicare claims from over 40 million Medicare beneficiaries. PMACS requires all users of data or applications on PMACS servers to complete a PMACS-hosted cybersecurity awareness course annually, which stresses federal data security policies under data use agreements with the university. The curriculum includes Health Insurance Portability and Accountability Act (HIPAA) training and covers secure data transfer, passwords, computer security habits and knowledge of what constitutes misuse or inappropriate use of the server. We will implement multiple, redundant protective measures to guarantee the privacy and security of the participant data. All investigators and research staff with direct access to the identifiable data will be required to undergo annual responsible conduct of research, cybersecurity, and HIPAA certification in accordance with University of Pennsylvania regulations. All data for this project will be stored on the secure/firewalled servers of the PMACS Data Center, in data files that will be protected by multiple password layers. These data servers are maintained in a guarded facility behind several locked doors, with very limited physical access rights. They are also cyber-protected by extensive firewalls and multiple layers of communication encryption. Electronic access rights are carefully controlled by University of Pennsylvania system managers. We will use highly secure methods of data encryption for all transactions involving participants' financial information using a level of security comparable to what is used in commercial financial transactions. We believe this multi-layer system of data security, identical to the system protecting the University of Pennsylvania Health Systems medical records, greatly minimizes the risk of loss of privacy. In addition, risk of loss of confidentiality will be minimized by storing completed paper copies of the surveys and signed informed consent forms in locked file cabinets in locked offices accessible only to trained study staff. Each subject will be assigned a unique identifier without identifying information, and data will be entered into an electronic database using only the unique identifier. Only trained study staff will have access to the code that links the unique identifier to the subject's identity. Electronic data will be stored on secure, password-protected firewalled servers at the University of Pennsylvania.

11.3 Subject privacy

Interested participants will be directed to the Way to Health portal where they will be asked to enter data related to eligibility and their demographic characteristics. Enrollment will include a description of the voluntary nature of participation, the study procedures, risks and potential benefits in detail. The enrollment procedure will provide the opportunity for potential participants to ask questions and review the consent form information with family and friends prior to making a decision to participate. Participants will be told that they do not have to answer any questions if they do not wish and can drop out of the study at any time, without affecting their medical care or the cost of their care. They will be told that they may or may not benefit directly from the study and that all information will be kept strictly confidential, except as

required by law. Subjects will be given a copy of the consent document. All efforts will be made by study staff to ensure subject privacy.

11.4 Data disclosure

The following entities, besides the members of the research team, may receive protected health information (PHI) for this research study: Wells Fargo, the company which processes study-related payments. Patient addresses and account balances will be stored on their secure computers. Withings, the company that designs and manufactures the wearable devices and weight scales used in the study to track participant physical activity. Twilio, Inc., the company which processes some study-related messages. Twilio will store patients' phone numbers on their secure computers. Qualtrics, Inc., the company which processes most study-related surveys. Qualtrics will house de-identified answers to these surveys on their secure servers. Quest, the laboratory used to measure hemoglobin A1c and LDL-C. The Office of Human Research Protections at the University of Pennsylvania -Federal and state agencies (for example, the Department of Health and Human Services, the National Institutes of Health, and/or the Office for Human Research Protections), or other domestic or foreign government bodies if required by law and/or necessary for oversight purposes.

11.5 Data safety and monitoring

The data monitoring system will track participant weight loss for the duration of the study. The electronic system will be programmed to alert the Study Coordinator by email whenever a participant loses more than 10 pounds in a week. Participants who are flagged for excessive weight loss will be contacted by phone by the research study coordinator. During this phone call the research study coordinator will discuss the importance of moderate weight loss rates (i.e., 1-2 pounds per week) with the participant, remind the participant that there is no additional monetary benefit to losing weight rapidly, and encourage the individual to moderate their weight loss habits. In addition, the coordinator will ask the participant if he or she is engaging in any behaviors such as self-induced vomiting or dehydration, fasting, or excessive exercise. All cases of excessive weight loss will be discussed with the PI, who will consider whether continuation in the study is appropriate based on patterns of excessive weight loss and information provided by the participant. For this study there will be no stopping rules or endpoints and thus no planned interim analyses.

11.6 Risk/benefit

11.6.1 Potential study risks

To minimize the chance for serious and unexpected adverse events, study participants will be screened through exclusion criteria for any health conditions that may be exacerbated by participating in a weight loss study. The intervention tries to motivate a gradual weight loss that should pose little health risk to participants. Another potential risk of this study is a breach of

participant confidentiality. We will minimize this risk by linking individual identifying information with participant ID numbers only in one single secure file that will only be accessed by the study team in the case of an adverse medical event, participant dropout, or if otherwise deemed necessary by the Principal Investigator. All other identifying information will be discarded after initial contact with the Study Coordinator. Due to the financial incentives in this study, we will be collecting social security numbers so that we can complete W-9 forms for participants. Social security numbers only will be used to generate W-9 forms and will be deleted once they are no longer needed. We will also collect home addresses to mail incentive payments. This will be done through a University of Pennsylvania approved partnership with Wells Fargo. Accidental disclosure of social security numbers could lead to identity theft. We will use commercial-grade encryption to protect social security information in transit. Names and addresses will be stored in encrypted databases. These data will be viewable only by the respective participants and the study coordinator. All other members of the research team will be able to view only participant ID numbers. There will be no functionality in the web application to export a dataset with identifiable information. Even the study arms will be identified by code letters until both the statistician and PI agree that analysis is complete.

11.6.2 Potential study benefits

Through participation in this study, each participant will have the potential to increase physical activity, lose weight, and improve glycemic control which could improve their health and reduce their risk for future disease. If this approach is effective, it could have tremendous benefits for society if adopted on a wide scale to help individuals with diabetes. It is expected that other people will gain knowledge from this study and that participation could help understand how to effectively motivate individuals to change behavior. Participants may also receive no benefit from their participation in the study.

11.6.3 Risk/benefit assessment

Anticipated risks of this study should be minimal and the risk/benefit ratio is very favorable. To minimize the chance for serious and unexpected adverse events, study participants will be screened through exclusion criteria for any health conditions that may be exacerbated by participating in this study. We have previously outlined the procedures that will be used to prevent a breach of participant data.

Final Study Protocol

A Randomized, Controlled Trial Using Social Incentives and Gamification to Change Health Behaviors and Improve Glycemic Control Among Type 2 Diabetics

Study Protocol

August 21, 2018

Outline

1. Abstract

2. Overall objectives

3. Aims

    3.1 Primary outcome

    3.2 Secondary outcomes

4. Background

5. Study design

    5.1 Design

    5.2 Study duration

    5.3 Target population

    5.4 Accrual

    5.5 Key inclusion criteria

    5.6 Key exclusion criteria

6. Subject recruitment

7. Subject compensation

8. Study procedures

    8.1 Consent

    8.2 Procedures

9. Analysis plan

10. Investigators

11. Human research protection

    11.1 Data confidentiality

    11.2 Subject confidentiality

    11.3 Subject privacy

11.4 Data disclosure

11.5 Data safety and monitoring

11.6 Risk/benefit

11.6.1 Potential study risks

11.6.2 Potential study benefits

11.6.3 Risk/benefit assessment

1. Abstract

Type 2 diabetes is a significant cause of morbidity and mortality in the United States. There is strong evidence that lifestyle modification including increasing physical activity and losing weight are effective for improving glycemic control. Insights from behavioral economics have shown promise for motivating behavior change through the use of financial incentives. However, social incentives or those influences that impact individuals to adjust their inherent behaviors based on social ties and connections have not been well examined. Social incentives are a more patient-centered approach that leverages and enhances the existing connections and influences on the individual. Since one individual's behavior change is connected to many others within their network, social incentives have the potential to be a scalable intervention that impacts population health. Gamification, or the use of game design in non-game situations, is often used in the real world, but social incentives are not often incorporated in the approach. In this study, we will conduct a one-year, four-arm, randomized, controlled trial to compare three social incentive-based gamification interventions to control. Each intervention will use insights from behavioral economics to leverage social incentives and gamification.

2. Overall objectives

The objective of this study is to use a randomized, controlled trial to test the effectiveness of three interventions using social incentives and gamification to promote physical activity, weight loss, and improved glycemic control among adult type 2 diabetics.

3. Aims

3.1 Primary outcome

The primary outcome variables are change in mean daily steps, weight in pounds, and hemoglobin A1c from baseline to end of the one-year study.

3.2 Secondary outcome

Secondary outcomes include the change in LDL-C levels from baseline to end of the one-year study period, as well as change in mean daily steps, weight in pounds, hemoglobin A1c, and LDL-C from baseline to the six-month midpoint of the study period.

4. Background

Type 2 diabetes is a significant cause of morbidity and mortality in the United States (1-2). There is strong evidence that lifestyle modification including increasing physical activity and losing weight are effective for improving glycemic control (3-4). However, there are several important reasons why these interventions are challenging to accomplish at broader scale. First, they involve behaviors that occur mostly outside of clinical encounters and instead within the everyday lives of patients (5). Second, past interventions targeting these behaviors have been

more personnel-intensive and therefore they are costly and complex to deploy at larger scale (5). Third, most interventions are based on the premise that individuals behave rationally and that if they are educated on the reasons for improving their health, that they will change their behaviors accordingly. However, we know that individuals do not behave in this manner (6) For example, many people know that physical inactivity and obesity is not good for their health, yet they don't regularly exercise or change their diet for weight loss. In the United States, more than 50% of adults do not obtain enough physical activity and more than 70% of adults are overweight or obese (7-8). Therefore, there is a significant need for new interventions that can passively collect data on daily health behaviors and combine automated feedback with behavioral strategies that anticipate and leverage predictable barriers to behavior change.

Behavioral economics is a field of study that incorporates principles from psychology to help us understand why individuals make decisions that are not aligned with their longer-term health goals. Instead, people often deviate from these goals in a predictable manner and from a common set of decision errors (6). For example, people tend to be more motivated by immediate rather than delayed gratification (9), by losses rather than gains (10), and they tend to take actions to avoid the feeling of regret (11). These insights have been incorporated in financial incentive-based approaches designed by our research group and resulted in improvements in weight loss (12-14), physical activity (15-17), smoking cessation (18-19), and adherence to remote-monitoring devices that collect patient-generated health data (20).

While financial incentives designed using behavioral economics have demonstrated success in motivating behavior change, social incentives or those influences that impact individuals to adjust their behaviors based on social ties and connections have not been well examined. Since each individual's behavior change is connected to many others within their network (21-22), social incentives have the potential to be a scalable intervention that impacts population health. There is evidence suggesting that social incentives can be very powerful in influencing behavior. For example, in a large retrospective study an individual's chances of becoming obese was 57% higher if a close friend became obese, 40% higher if a sibling became obese, and 37% higher if a spouse became obese (21). In comparison, when an individual's spouse or partner loses weight, the individual is three times more likely to also lose weight (23). Therefore, there is a significant opportunity to improve health by better understanding how to design social incentive interventions to motivate behavior change.

[1] Gregg EW, Li Y, Wang J, Burrows NR, Ali MK, Rolka D, Williams DE, Geiss L. Changes in diabetes-related complications in the United States, 1990-2010. N Engl J Med. 2014;370(16):1514-23. [2] Menke A, Casagrande S, Geiss L, Cowie CC. Prevalence of and Trends in Diabetes Among Adults in the United States, 1988-2012. JAMA. 2015;314(10):1021-9. [3] Pronk NP, Remington PL. Combined Diet and Physical Activity Promotion Programs for Prevention of Diabetes: Community Preventive Services Task Force Recommendation Statement. Ann Intern Med. 2015;163(6):465-469. [4] Jeon CY, Lokken RP, Hu FB, van Dam RM. Physical activity of moderate intensity and risk of type 2 diabetes: a systematic review.

Diabetes Care. 2007;30(3):744-52. [5] Asch DA, Muller RW, Volpp KG. Automated hovering in health care--watching over the 5000 hours. N Engl J Med. 2012;367(1):1-3. [6] Loewenstein G, Brennan T, Volpp KG. Asymmetric paternalism to improve health behaviors. JAMA. 2007;298(20):2415-7. [7] Centers for Disease Control and Prevention. Facts about physical activity. Accessed at www.cdc.gov/physicalactivity/data/facts.html [8] Ogden CL, Carroll MD, Kit BK, Flegal KM. Prevalence of childhood and adult obesity in the United States, 2011–2012. JAMA. 2014;311(8): 806–14. [9] O'Donoghue T, Rabin M. The economics of immediate gratification. Journal of Behavioral Decision Making. 2000;13:233-250. [10] Kahneman D, Tversky A. Prospect theory: an analysis of decision under risk. Econometrica. 1979;47:263-91. [11] Zeelenberg M, Pieters R. Consequences of regret aversion in real life: the case of the Dutch postcode lottery. Organ Behav Hum Decis Process. 2004;93:155-68. [12] Volpp KG, John LK, Troxel AB, Norton L, Fassbender J, Loewenstein G. Financial incentive-based approaches for weight loss: a randomized trial. JAMA. 2008;300(22):2631-7. [13] John LK, Loewenstein G, Troxel AB, Norton L, Fassbender JE, Volpp KG. Financial incentives for extended weight loss: a randomized, controlled trial. J Gen Intern Med. 2011:26(6):621–626. [14] Kullgren JT, Troxel AB, Loewenstein G, Asch DA, Norton LA, Wesby L, Tao Y, Zhu J, Volpp KG. Individual-versus group-based financial incentives for weight loss: a randomized, controlled trial. Ann Intern Med. 2013;158(7):505-14. [15] Patel MS, Asch DA, Rosin R, Small DS, Bellamy SL, Heuer J, Sproat S, Hyson C, Haff N, Lee SM, Wesby L, Hoffer K, Shuttleworth D, Taylor D, Hilbert V, Zhu J, Yang L, Wang X, Volpp KG. Framing financial incentives to increase physical activity among overweight and obese adults: a randomized, controlled trial. Ann Intern Med. 2016;164(6):385-396. [16] Patel MS, Asch DA, Rosin R, Small DS, Bellamy SL, Eberbach K, Walters KJ, Haff N, Lee SM, Wesby L, Hoffer K, Shuttleworth D, Taylor D, Hilbert V, Zhu J, Yang L, Wang X, Volpp KG. Individual versus team-based financial incentives to increase physical activity: a randomized, controlled trial. J Gen Intern Med. . 2016;Published Online March 14, 2016. DOI: 10.1007/s11606-016-3627-0. [17] Patel MS, Volpp KG, Rosin R, Bellamy SL, Small DS, Fletcher MA, Osman-Koss R, Brady JL, Haff N, Lee SM, Wesby L, Hoffer K, Shuttleworth D, Taylor DH, Ulrich V, Zhu J, Yang L, Wang X, Asch DA. A Randomized Trial of Social Comparison Feedback and Financial Incentives to Increase Physical Activity. Am J Health Promot. In Press. [18] Volpp KG, Troxel AB, Pauly MV, Glick HA, Puig A, Asch DA, Galvin R, Zhu J, Wan F, DeGuzman J, Corbett E, Weiner J, Audrain-McGovern J. A randomized, controlled trial of financial incentives for smoking cessation. N Engl J Med. 2009;360(7):699-709. [19] Halpern SD, French B, Small DS, Saulsgiver K, Harhay MO, Audrain-McGovern J, Loewenstein G, Brennan TA, Asch DA, Volpp KG. Randomized trial of four financial-incentive programs for smoking cessation. N Engl J Med. 2015;372(22):2108-17. [20] Sen AP, Sewell TB, Riley EB, Stearman B, Bellamy SL, Hu MF, Tao Y, Zhu J, Park JD, Loewenstein G, Asch DA, Volpp KG. Financial incentives for home-based health monitoring: a randomized controlled trial. J Gen Intern Med. 2014;29(5):770-7. [21] Christakis NA, Fowler JH. The spread of obesity in a large social network over 32 years. N Engl J Med. 2007;357(4):370-9. [22] Christakis NA, Fowler JH. The collective dynamics of smoking in a

large social network. N Engl J Med. 2008;358(21):2249-58. [23] Jackson SE, Steptoe A, Wardle J. The Influence of Partner's Behavior on Health Behavior Change: The English Longitudinal Study of Ageing. JAMA Intern Med. 2015;175(3):385-92.

5. Study design

5.1 Design

This is a four-arm, one-year randomized, controlled trial. The study will be conducted using Way to Health, an automated information technology platform at the University of Pennsylvania that integrates wireless devices, conducts clinical trial randomization and enrollment processes, delivers messaging (text or email) delivers self-administered surveys, automates payment transfers, and securely captures data for research purposes.

The study team will identify potential participants from the electronic health record at the University of Pennsylvania Health System using Penn Data Store and Clarity, an EPIC reporting database. Patients with a hemoglobin A1c of 8.0 or greater and body mass index of 25 or greater will receive either an email, letter, phone call, or some combination from the study team introducing the study and its eligibility criteria with instructions on how to learn more and begin the enrollment process. Interested participants will be instructed to visit the study website to create an account, review and complete informed consent, and complete eligibility survey. Eligible participants will be instructed to obtain a hemoglobin A1c and LDL-C laboratory test, unless they already have recent values (within 5 weeks) in EPIC which qualify them for the study. In this case, the value from EPIC will be used so the patient doesn’t have to go to a laboratory to get their labs done again. If the hemoglobin A1c is 8.0 or greater and the participant is still interested in continuing the enrollment process, a wearable activity tracking device will be mailed to them to wear for about two weeks to collect a baseline step count. After that, the participant will be scheduled for an in-person visit with the study team to complete the enrollment process including surveys. During this process the participant will be told that they may be randomized to participate individually or on a team with up to two other members. If they have a family member, friend, or coworker that might be interested in also participating, they can let the study team know that they would like to be randomized together.

During the in-person visit, all participants will receive education on the importance of diet and physical activity for weight loss and glycemic control. Each participant will have already received a wearable activity tracker and will be informed that he or she should wear this device during the one-year study period. Each participant will be giving a wireless weight scale for monitoring their weight at home. They will establish a baseline weight measure at the in-person visit. Since weight measures may vary based on time of day and in relation to last meal, we will monitor their first weight at home and if 5 lbs. or greater different will call the patient to assess which weight is closer to their true weight and use that as their baseline.

Participants will be randomly assigned in blocks of four groups to one of the four study arms stratified based on whether the group of individuals already knew each other or not. Groups will have three individuals unless the individuals were already socially connected prior to the study, in which case they will be allowed to be randomized as a group of two or three. Participants randomized to the control arm will receive no other interventions.

--Goal Setting--

Each participant in the intervention arms will be asked to choose their goals for the study as follows: 1) Choose a weight loss goal that is 6%, 7%, or 8% of their baseline weight rounded up to the next pound; 2) Choose a daily step count goal that is 33%, 40%, or 50% greater than their baseline rounded up to the nearest hundred, or choose their own step goal as long as it is at least 1500 steps above their baseline; 3) Choose a HbA1c reduction goal of 1.5%, 2%, or 2.5%.

The weight loss goal will be set for a gradual decline over 26 weeks. The participant is given a weight target for each week in the study and if not achieved, then the target remains the same for the following week.

Participants will have a 4-week ramp up towards their step goal. The net difference between baseline and their goal will be divided by 4 and the participant will be asked to achieve the 25% increase each week for 4-week and then maintain the step goal for the remaining study period. For example, a participant with a baseline of 6000 steps and goal of 8000 steps will be asked to achieve goals of 6500, 7000, 7500, and 8000 for each of the first four weeks of the study. The they will be asked to maintain the 8000 step goal.

The HbA1c goal will be set for the 6-month mark and be expected to be maintained through the one-year mark.

At 3, 6, and 9 months, participants struggling to meet their goals (defined as in the blue or bronze levels of the game, see below) will be called to inquire about their progress in the study, moved up to the silver level, and given the opportunity to readjust their goals among the choices above.

All participants will receive $25 to complete bloodwork during the enrollment process, whether they are determined to be eligible or ineligible from the results. Participants who are eligible and complete enrollment into the study (defined as being randomized and starting the intervention) will receive an additional $25. Participants will receive $50 to obtain lab tests (HbA1c and LDL-C) and conduct a virtual weigh-in at home by facetime or skype after 6 months and $50 to obtain lab tests (HbA1c and LDL-C) and conduct a virtual weigh-in at home by facetime or skype after one year.

--Social Incentive Interventions (Arms 2-4)--

Participants in arms 2-4 will be entered into an intervention approach that has points and levels designed to incorporate insights from behavioral economics. On a daily basis, they will be asked

to weigh-in.   They will have a weekly weight target and mean step goal. First, participants will be asked to sign a pre-commitment pledge to strive to achieve their goals during the study. Pre-commitment has been demonstrated to motivate behavior change. Second, at the beginning of each week the participant receives 70 points (10 for each day that week). If the participant does not weigh-in, they lose 10 points from their balance. This leverages loss aversion, which has been demonstrated to motivate behavior change more effectively with losses than gains. Third, at the end of each week if you have at least 40 points, met your weekly weight and step goal, you will move up a level (levels from lowest to highest: blue, bronze, silver, gold, platinum). If not, participants will drop a level. All participants begin at the silver level. Each week, participants get a fresh set of 70 points on Monday.

Weekly feedback will differ among the three arms to induce the different social incentives (support, competition, and collaboration) as follows:

Arm 2 – Supportive social incentive intervention: Participants in this arm will be asked to identify a family member or friend to be their support sponsor. A weekly report will be sent to this person with the participant's performance (goal achievement, points and level). A monthly report will be sent to the participant's primary care physician (PCP) with data on their change in weight, step goals, HbA1c, and LDL-C. A copy of this letter will be sent to the participant. The sponsor and PCP will serve as part of the participant's support network and be encouraged to reach out to the participant.

Arm 3 – Competitive social incentive intervention: Participants in this arm will be in a group of two or three people. At the end of each week the participants will receive an email with a leaderboard. In the leaderboard, group members will be ranked first by current level and then by their total points to date. This feedback may help to induce participants to compete for the top spot among the group. A monthly report will be sent to the participant's primary care physician (PCP) with data on their change in weight, step goals, HbA1c, and LDL-C.   A copy of this letter will be sent to the participant.

Arm 4 – Collaborative social incentive intervention: Participants in this arm will be in a group of two or three people. Each day one of members of the group will be randomly selected to represent their team for that day. If they met their goal on the previous day (e.g. weighed in), everyone on the team keeps their points. If they didn't meet their goal then everyone on the team loses 10 points. In this design, each person is accountable to the others on the team and this may induce a collaborative effort to meet their daily goals. The entire team moves up a level only if all team members met their weekly weight and step targets. A monthly report will be sent to the participant's primary care physician (PCP) with data on their change in weight, step goals, HbA1c, and LDL-C. A copy of this letter will be sent to the participant.

Participants will have the opportunity to win a trophy, plaque, or medal depending on their level at the end of the study.

5.2 Study duration

This is one-year study.

5.3 Target population

Adults type 2 diabetics age 18 years to 70 years with a body mass index of 25 or greater and a HbA1c of 8 or greater.

5.4 Accrual

We estimate that a sample size of 360 participants (90 per arm) will provide at least 80% power using a conservative Bonferroni adjustment of the Type I error rate with a 2-sided α of 0.017 and accounting for a dropout rate of 10% to detect: 1) a 1100 step change in physical activity with a standard deviation of 2000 steps; 2) a 6 lb. change in weight with a standard deviation of 10 lbs; and 3) a 0.8% change in HbA1c with a standard deviation of 1.5%.

5.5 Key inclusion criteria

1) Age 18 years to 70 years; 2) ability to read and provide informed consent to participate in the study; 3) diagnosis of type 2 diabetes with a hemoglobin A1c of 8.0 or greater; 4) Self-reported body mass index (BMI) of 25 or greater.; 5) Smartphone or tablet compatible with the application for the wearable activity tracking device and wireless weight scale.

5.6 Key exclusion criteria

1) Conditions that would make participation infeasible such as inability to provide informed consent, illiteracy or inability to speak, read, and write English; 2) conditions that would make participation unsafe such as pregnancy, previous diagnosis of an eating disorder, or history of unsafe weight loss practices; 3) already enrolled in another study targeting physical activity, weight loss, or glycemic control; 4) any other medical conditions or reasons he or she is unable to participate in the study for one year.

6. Subject recruitment

The study team will identify potential participants from the electronic health record at the University of Pennsylvania Health System using Penn Data Store and Clarity, an EPIC reporting database. Patients with a hemoglobin A1c of 8.0 or greater and body mass index of 25 or greater will receive either an email, letter, phone call, or some combination from the study team introducing the study and its eligibility criteria with instructions on how to learn more and begin the enrollment process. We may also post flyers in UPHS primary care facilities and endocrinologist clinics.

7. Subject compensation

All participants will receive $25 to complete bloodwork during the enrollment process, whether they are determined to be eligible or ineligible from the results. Participants who are eligible and complete enrollment in the study (defined as being randomized and starting the intervention) will receive an additional $25. Participants will recieve $50 to obtain lab tests (HbA1c and LDL-C) and conduct a virtual weigh-in at home by facetime or skype after 6 months and another $50 to obtain lab tests (HbA1c and LDL-C) and conduct a virtual weigh-in at home by facetime or skype after one year.

8. Study procedures

8.1 Consent

Upon recruitment, individuals who are interested in learning more about the study will be directed to the Way to Health web portal. Upon reaching the portal, potential participants will be asked to create an account and will then be informed of the details of the study, including its objectives, duration, requirements, and financial payments. If participants are still interested in participating, the Way to Health portal will take them through an automated online informed consent. The consent document will be divided into sections and potential participants will have to click a button to advance through each section. This is to help ensure that participants read the consent form thoroughly by breaking down the form into manageable blocks of text. Each section will have a button allowing the user to contact a researcher via email or by telephone if they have questions about the consent form. Successive screens will explain the voluntary nature of the study, the risks and benefits of participation, alternatives to participation, and that participants can withdraw from the study at any time. On the final consent screen, potential participants who click a clearly delineated button stating that they agree to participate in the study will be considered to have consented to enroll. Participants will be provided with details regarding how to contact the research team via email or phone at any time if they subsequently wish to withdraw from the study. This contact information will remain easily accessible via the participants' individual Way to Health web portal dashboards throughout the study. When the participant conducts the in-person meeting with the study team, they will be asked to physically sign a copy of the informed consent.

8.2 Procedures

After providing informed consent, participants will complete an online questionnaire to determine their eligibility and complete the study surveys. Eligible participants will be instructed to obtain a hemoglobin A1c and LDL-C laboratory test, unless they already have recent values (within 5 weeks) in EPIC which qualify them for the study. In this case, the value from EPIC will be used so the patient doesn't have to go to a laboratory to get their labs done again. These laboratory test measure the body's glucose and cholesterol control and will require no more than two vials of blood drawn. During the study, participants will be asked to obtain these measures at baseline and after 6 and 12 months. If the hemoglobin A1c is 8.0 or greater at baseline and the

participant is still interested in continuing the enrollment process, a wearable activity tracking device will be mailed to them to wear for about two weeks to collect a baseline step count. After that, the participant will be scheduled for an in-person visit with the study team to complete the enrollment process. During this process the participant will be told that they may be randomized to participate individually or on a team with up to two other members. If they have a family member, friend, or coworker that might be interested in also participating, they can let the study team know that they would like to be randomized together.

During the in-person visit, all participants will receive education on the importance of diet and physical activity for weight loss and glycemic control. Each participant will have already received a wearable activity tracker and will be informed that he or she should wear this device all the time (during the day and while sleeping) during the one-year study period. Each participant will be giving a wireless weight scale for monitoring their weight at home. They will establish a baseline weight measure at the in-person visit. Since weight measures may vary based on time of day and in relation to last meal, we will monitor their first weight at home and if more than 5 lbs. different will call the patient to assess which weight is closer to their true weight and use that as their baseline.

Participants will be randomly assigned in blocks of four groups to one of the four study arms stratified based on whether the group of individuals already knew each other or not. Groups will have three individuals unless the individuals were already socially connected prior to the study, in which case they will be allowed to be randomized as a group of two or three. Participants randomized to the control arm will receive no other interventions.

9. Analysis plan

To compare sample characteristics between arms we will use t-tests or Wilcoxon rank-sum tests (F-tests or Kruskal-Wallis test) for continuous variables and Pearson chi square tests or Fisher's exact tests for categorical variables. In our primary analyses, we will compare change in mean daily steps (baseline vs. one-year study period), weight in pounds (baseline vs. end of one-year study period), and hemoglobin A1c (baseline vs. end of one-year study period) for each intervention arm compared to the control arm. In the secondary analysis we will compare change in LDL-C from baseline to the end of one-year study period. For each of the four measures listed we will also compare outcomes after 6-months. We will compare proportion of time the step goals were achieved during the one-year study. Our primary analyses will use multiple imputation for step data that are missing and for missing weight measures or missing hemoglobin A1c measures. We will conduct a series of adjusted regression models that include controlling for baseline measures, calendar month fixed effects, repeated measures and other participant characteristics such as demographics, comorbidities, self-reported measures, and validated survey measures. All hypothesis tests will be two-sided using a two-sided alpha of 0.017 as our threshold for statistical significance. We will use Stata and/or SAS to analyze the data.

We will conduct exploratory analyses on differences in sleep measures such as duration and quality and differences in heart rate measures such as baseline, average, and peak. We will conduct an exploratory qualitative evaluation of the survey free responses.

10. Investigators

Mitesh Patel, MD, MBA, MS is the Principal Investigator (PI) and is an Assistant Professor of Medicine and Health Care Management at the Perelman School of Medicine and The Wharton School at the University of Pennsylvania. He has past experience leading randomized, clinical trials using the Way to Health Platform to deploy interventions using financial and social incentives to promote weight loss and increased physical activity. He currently spends 80% of his effort on research and 20% on clinical and teaching activities.

11. Human research protection

11.1 Data confidentiality

Paper-based records will be kept in a secure location and only be accessible to personnel involved in the study. Computer-based files will only be made available to personnel involved in the study through the use of access privileges and passwords. Wherever feasible, identifiers will be removed from study-related information. Precautions are in place to ensure the data are secure by using passwords and encryption, because the research involves web-based surveys.

11.2 Subject confidentiality

Research material will be obtained from participant surveys, laboratory testing, wearable devices, and weight scales. All participants will provide informed consent for access to these materials. The data to be collected include demographic data (e.g., age, sex, self-identified race), hemoglobin A1c, LDL-C, step, sleep, and weight data. Research material that is obtained will be used for research purposes only. The same procedure used for the analysis of automated data sources to ensure protection of patient information will be used for the survey data, in that patient identifiers will be used only for linkage purposes or to contact patients. The study identification number, and not other identifying information, will be used on all data collection instruments. All study staff will be reminded to appreciate the confidential nature of the data collected and contained in these databases. The Penn Medicine Academic Computing Services (PMACS) will be the hub for the hardware and database infrastructure that will support the project and is where the Way to Health web portal is based. The PMACS is a joint effort of the University of Pennsylvania's Abramson Cancer Center, the Cardiovascular Institute, the Department of Pathology, and the Leonard Davis Institute. The PMACS provides a secure computing environment for a large volume of highly sensitive data, including clinical, genetic, socioeconomic, and financial information. Among the IT projects currently managed by PMACS are: (1) the capture and organization of complex, longitudinal clinical data via web and clinical applications portals from cancer patients enrolled in clinical trials; (2) the integration of genetic

array databases and clinical data obtained from patients with cardiovascular disease; (3) computational biology and cytometry database management and analyses; (4) economic and health policy research using Medicare claims from over 40 million Medicare beneficiaries. PMACS requires all users of data or applications on PMACS servers to complete a PMACS-hosted cybersecurity awareness course annually, which stresses federal data security policies under data use agreements with the university. The curriculum includes Health Insurance Portability and Accountability Act (HIPAA) training and covers secure data transfer, passwords, computer security habits and knowledge of what constitutes misuse or inappropriate use of the server. We will implement multiple, redundant protective measures to guarantee the privacy and security of the participant data. All investigators and research staff with direct access to the identifiable data will be required to undergo annual responsible conduct of research, cybersecurity, and HIPAA certification in accordance with University of Pennsylvania regulations. All data for this project will be stored on the secure/firewalled servers of the PMACS Data Center, in data files that will be protected by multiple password layers. These data servers are maintained in a guarded facility behind several locked doors, with very limited physical access rights. They are also cyber-protected by extensive firewalls and multiple layers of communication encryption. Electronic access rights are carefully controlled by University of Pennsylvania system managers. We will use highly secure methods of data encryption for all transactions involving participants' financial information using a level of security comparable to what is used in commercial financial transactions. We believe this multi-layer system of data security, identical to the system protecting the University of Pennsylvania Health Systems medical records, greatly minimizes the risk of loss of privacy. In addition, risk of loss of confidentiality will be minimized by storing completed paper copies of the surveys and signed informed consent forms in locked file cabinets in locked offices accessible only to trained study staff. Each subject will be assigned a unique identifier without identifying information, and data will be entered into an electronic database using only the unique identifier. Only trained study staff will have access to the code that links the unique identifier to the subject's identity. Electronic data will be stored on secure, password-protected firewalled servers at the University of Pennsylvania.

11.3 Subject privacy

Interested participants will be directed to the Way to Health portal where they will be asked to enter data related to eligibility and their demographic characteristics. Enrollment will include a description of the voluntary nature of participation, the study procedures, risks and potential benefits in detail. The enrollment procedure will provide the opportunity for potential participants to ask questions and review the consent form information with family and friends prior to making a decision to participate. Participants will be told that they do not have to answer any questions if they do not wish and can drop out of the study at any time, without affecting their medical care or the cost of their care. They will be told that they may or may not benefit directly from the study and that all information will be kept strictly confidential, except as

required by law. Subjects will be given a copy of the consent document. All efforts will be made by study staff to ensure subject privacy.

11.4 Data disclosure

The following entities, besides the members of the research team, may receive protected health information (PHI) for this research study: Wells Fargo, the company which processes study-related payments. Patient addresses and account balances will be stored on their secure computers. Nokia, the company that designs and manufactures the wearable devices and weight scales used in the study to track participant physical activity. Twilio, Inc., the company which processes some study-related messages. Twilio will store patients' phone numbers on their secure computers. Qualtrics, Inc., the company which processes most study-related surveys. Qualtrics will house de-identified answers to these surveys on their secure servers. Quest, the laboratory used to measure hemoglobin A1c and LDL-C. The Office of Human Research Protections at the University of Pennsylvania -Federal and state agencies (for example, the Department of Health and Human Services, the National Institutes of Health, and/or the Office for Human Research Protections), or other domestic or foreign government bodies if required by law and/or necessary for oversight purposes.

11.5 Data safety and monitoring

The data monitoring system will track participant weight loss for the duration of the study. The electronic system will be programmed to alert the Study Coordinator by email whenever a participant loses more than 10 pounds in a week. Participants who are flagged for excessive weight loss will be contacted by phone by the research study coordinator. During this phone call the research study coordinator will discuss the importance of moderate weight loss rates (i.e., 1-2 pounds per week) with the participant, remind the participant that there is no additional monetary benefit to losing weight rapidly, and encourage the individual to moderate their weight loss habits. In addition, the coordinator will ask the participant if he or she is engaging in any behaviors such as self-induced vomiting or dehydration, fasting, or excessive exercise. All cases of excessive weight loss will be discussed with the PI, who will consider whether continuation in the study is appropriate based on patterns of excessive weight loss and information provided by the participant. For this study there will be no stopping rules or endpoints and thus no planned interim analyses.

11.6 Risk/benefit

11.6.1 Potential study risks

To minimize the chance for serious and unexpected adverse events, study participants will be screened through exclusion criteria for any health conditions that may be exacerbated by participating in a weight loss study. The intervention tries to motivate a gradual weight loss that should pose little health risk to participants. Another potential risk of this study is a breach of

participant confidentiality. We will minimize this risk by linking individual identifying information with participant ID numbers only in one single secure file that will only be accessed by the study team in the case of an adverse medical event, participant dropout, or if otherwise deemed necessary by the Principal Investigator. All other identifying information will be discarded after initial contact with the Study Coordinator. Due to the financial incentives in this study, we will be collecting social security numbers so that we can complete W-9 forms for participants. Social security numbers only will be used to generate W-9 forms and will be deleted once they are no longer needed. We will also collect home addresses to mail incentive payments. This will be done through a University of Pennsylvania approved partnership with Wells Fargo. Accidental disclosure of social security numbers could lead to identity theft. We will use commercial-grade encryption to protect social security information in transit. Names and addresses will be stored in encrypted databases. These data will be viewable only by the respective participants and the study coordinator. All other members of the research team will be able to view only participant ID numbers. There will be no functionality in the web application to export a dataset with identifiable information. Even the study arms will be identified by code letters until both the statistician and PI agree that analysis is complete.

11.6.2 Potential study benefits

Through participation in this study, each participant will have the potential to increase physical activity, lose weight, and improve glycemic control which could improve their health and reduce their risk for future disease. If this approach is effective, it could have tremendous benefits for society if adopted on a wide scale to help individuals with diabetes. It is expected that other people will gain knowledge from this study and that participation could help understand how to effectively motivate individuals to change behavior. Participants may also receive no benefit from their participation in the study.

11.6.3 Risk/benefit assessment

Anticipated risks of this study should be minimal and the risk/benefit ratio is very favorable. To minimize the chance for serious and unexpected adverse events, study participants will be screened through exclusion criteria for any health conditions that may be exacerbated by participating in this study. We have previously outlined the procedures that will be used to prevent a breach of participant data.
