# Supplementary material for: Effect of Behaviorally Designed Gamification With Social Incentives on Lifestyle Modification Among Adults With Uncontrolled Diabetes: A Randomized Clinical Trial
Source: JAMA Netw Open. 2021 May 24;4(5):e2110255. doi: 10.1001/jamanetworkopen.2021.10255 (PMC8144928; doi:10.1001/jamanetworkopen.2021.10255)
Supplement: Supplement 2. — eTable 1. Missing Data Rates by Arm and Study Period eTable 2. Number of Observations Collected by Outcome and Time Period eTable 3. Adjusted Differences in Outcomes Without Multiple Imputation eTable 4. Patient Perceptions of Trial Experience From End of Study Surveys [file jamanetwopen-e2110255-s002.pdf]

Supplementary Online Content

Patel MS, Small DS, Harrison JD, et al. Effect of behaviorally designed gamification with social incentives on lifestyle modification among adults with uncontrolled diabetes: a randomized clinical trial. *JAMA Netw Open*. 2021;4(5):e2110255. doi:10.1001/jamanetworkopen.2021.10255

eTable 1. Missing Data Rates by Arm and Study Period

eTable 2. Number of Observations Collected by Outcome and Time Period

eTable 3. Adjusted Differences in Outcomes Without Multiple Imputation

eTable 4. Patient Perceptions of Trial Experience From End of Study Surveys

This supplementary material has been provided by the authors to give readers additional information about their work.

© 2021 Patel MS et al. *JAMA Network Open.*

eTable 1: Missing data rates by arm and study period.

Outcome	Control	Gamification with Support	Gamification with Collaboration	Gamification with Competition
**Steps per Day**				
**Months 1-12**	12862/31755 (40.5%)	10938/33580 (32.6%)	9052/34675 (26.1%)	10766/31755 (33.9%)
**Weight**				
**Month 6**	8/87 (9.2%)	4/92 (4.3%)	2/95 (2.1%)	5/87 (5.7%)
**Month 12**	22/87 (25.3%)	8/92 (8.7%)	4/95 (4.2%)	9/87 (10.3%)
**Hemoglobin A1c**				
**Month 6**	18/87 (20.7%)	17/93 (18.3%)	20/95 (21.1%)	21/87 (24.1%)
**Month 12**	27/87 (31%)	27/93 (29%)	23/95 (24.2%)	28/87 (32.2%)
**LDL Cholesterol**				
**Month 6**	27/87 (31%)	30/92 (32.6%)	32/95 (33.7%)	33/87 (37.9%)
**Month 12**	32/87 (36.8%)	41/92 (44.6%)	38/95 (40%)	40/87 (46%)

Step data is presented at the participant-day level. For example, in control there were 87 patients and therefore 87 patients x 365 days = 31755 participant-days for that period.

© 2021 Patel MS et al. *JAMA Network Open.*

eTable 2: Number of observations collected by outcome and time period

Outcome	Timepoint	Control	Gamification with Support	Gamification with Collaboration	Gamification with Competition
Physical Activity, Steps per Day	**Months 1-6, N**	10377	12459	13463	11210
	**Months 1-12, N**	16452	20890	23046	19190
Weight, lbs.	**Month 6, N**	79	88	93	82
	**Month 12, N**	65	84	91	78
Hemoglobin A1c	**Month 6, N**	69	76	75	66
	**Month 12, N**	60	66	72	57
LDL Cholesterol	**Month 6, N**	60	62	63	54
	**Month 12, N**	55	51	57	47

© 2021 Patel MS et al. *JAMA Network Open.*

eTable 3: Adjusted differences in outcomes without multiple imputation

Outcome	Timepoint	Control	Gamification with Support	Gamification with Collaboration	Gamification with Competition
Physical Activity, Steps per Day	**Baseline, Mean (SD)**	4410 (2263)	4353 (2439)	4122 (2817)	4681 (2893)
	**Months 1-6, Mean (SD)**	4143 (2416)	4857 (2647)	4391 (2726)	5071 (3059)
	Difference relative to control (95% CI)	-	727 (263, 1191)	491 (32, 950)	677 (207,1146)
	P Value	-	0.002	0.04	0.005
	**Months 1-12, Mean (SD)**	4068 (2318)	4649 (2505)	4068 (2318)	4915 (295)
	Difference relative to control (95% CI)	-	594 (113, 1055)	349 (-107, 805)	616 (149, 1082)
	P Value	-	0.01	0.13	0.01
Weight, lbs.	**Baseline, Mean (SD)**	235.5 (49.6)	227.9 (40.2)	244.3 (47.2)	238.8 (45.6)
	**Month 6, Mean (SD)**	226.3 (46.7)	220.2 (39.6)	238.6 (47.6)	232.2 (45.2)
	Difference relative to control (95% CI)	-	-3.1 (-6.8, 0.6)	0.0 (-3.7, 3.7)	1.5 (-2.2, 5.3)
	P Value	-	0.10	0.98	0.42
	**Month 12, Mean (SD)**	231.5 (53.2)	219.9 (40.4)	236.4 (46.7)	229.9 (46.2)
	Difference relative to control (95% CI)	-	-4.9 (-10.2, 0.4)	-3.6 (-8.8, 1.6)	-2.5 (-7.8, 2.9)
	P Value	-	0.07	0.18	0.37
Hemoglobin A1c	**Baseline, Mean (SD)**	9.5 (1.5)	9.5 (1.5)	9.9 (1.7)	9.7 (1.6)
	**Month 6, Mean (SD)**	8.7 (1.7)	9.0 (1.9)	8.8 (2.0)	8.7 (1.7)
	Difference relative to control (95% CI)	-	0.25 (-0.30, 0.80)	-0.19 (-0.74, 0.37)	-0.14 (-0.72, 0.43)
	P Value	-	0.38	0.51	0.63
	**Month 12, Mean (SD)**	8.8 (1.8)	8.9 (1.9)	8.8 (2.2)	8.7 (2.0)
	Difference relative to control (95% CI)	-	0.09 (-0.59, 0.77)	-0.19 (-0.86, 0.48)	-0.21 (-0.89, 0.47)
	P Value	-	0.80	0.58	0.55
LDL Cholesterol	**Baseline, Mean (SD)**	99.9 (31.2)	101.7 (41.8)	97.9 (32.4)	90.0 (38.7)
	**Month 6, Mean (SD)**	91.9 (36.6)	95.1 (37.5)	92.9 (34.1)	82.5 (38.1)
	Difference relative to control (95% CI)	-	1.6 (-7.9, 11.1)	-0.9 (-10.3, 8.6)	0.3 (-9.6, 10.2)
	P Value	-	0.73	0.86	0.95
	**Month 12, Mean (SD)**	97.5 (36.3)	96.5 (34.8)	92.6 (31.7)	94.7 (38.0)
	Difference relative to control (95% CI)	-	-1.9 (-13.2, 9.4)	-6.3 (-17.3, 4.8)	-2.5 (-13.9, 9.0)
	P Value	-	0.75	0.27	0.67

© 2021 Patel MS et al. *JAMA Network Open.*

eTable 4: Patient perceptions of trial experience from end of study surveys

Survey Questions	**Control (n = 47)**	**Gamification with Support (n = 40)**	**Gamification with Collaboration (n = 44)**	**Gamification with Competition (n = 31)**
**This study helped me to increase my physical activity level, No. (%)**				
Strongly agree	15 (31.9%)	12 (30%)	18 (40.9%)	10 (32.3%)
Agree	18 (38.3%)	17 (42.5%)	18 (40.9%)	8 (25.8%)
Neutral	9 (19.1%)	6 (15%)	8 (18.2%)	12 (38.7%)
Disagree	4 (8.5%)	5 (12.5%)	0 (0%)	1 (3.2%)
Strongly disagree	1 (2.1%)	0 (0%)	0 (0%)	0 (0%)
**This study helped me to lose weight, No. (%)**				
Strongly agree	8 (17%)	10 (25%)	9 (20.9%)	5 (16.1%)
Agree	11 (23.4%)	11 (27.5%)	16 (37.2%)	12 (38.7%)
Neutral	19 (40.4%)	13 (32.5%)	12 (27.9%)	8 (25.8%)
Disagree	6 (12.8%)	5 (12.5%)	6 (14%)	6 (19.4%)
Strongly disagree	3 (6.4%)	1 (2.5%)	0 (0%)	0 (0%)
**This study helped me to improve my diabetes control, No. (%)**				
Strongly agree	10 (21.3%)	8 (20%)	12 (27.3%)	3 (9.7%)
Agree	11 (23.4%)	19 (47.5%)	17 (38.6%)	12 (38.7%)
Neutral	19 (40.4%)	8 (20%)	10 (22.7%)	11 (35.5%)
Disagree	6 (12.8%)	4 (10%)	5 (11.4%)	5 (16.1%)
Strongly disagree	1 (2.1%)	1 (2.5%)	0 (0%)	0 (0%)
**I was satisifed with my experience using the wearable device to track my daily step counts, No. (%)**				
Strongly agree	15 (31.9%)	16 (40%)	19 (43.2%)	8 (25.8%)
Agree	24 (51.1%)	18 (45%)	15 (34.1%)	15 (48.4%)
Neutral	5 (10.6%)	0 (0%)	7 (15.9%)	4 (12.9%)
Disagree	1 (2.1%)	2 (5%)	3 (6.8%)	3 (9.7%)
Strongly disagree	2 (4.3%)	4 (10%)	0 (0%)	1 (3.2%)
**I was satisifed with my experience using the weight scale to record and transmit my**				
Strongly agree	24 (51.1%)	23 (57.5%)	24 (54.5%)	13 (41.9%)
Agree	16 (34%)	12 (30%)	15 (34.1%)	12 (38.7%)
Neutral	6 (12.8%)	2 (5%)	2 (4.5%)	5 (16.1%)
Disagree	1 (2.1%)	0 (0%)	2 (4.5%)	1 (3.2%)
Strongly disagree	0 (0%)	3 (7.5%)	1 (2.3%)	0 (0%)
**Overall, I enjoyed participating in this study, No. (%)**				
Strongly agree	19 (40.4%)	22 (55%)	24 (55.8%)	10 (32.3%)
Agree	21 (44.7%)	10 (25%)	12 (27.9%)	14 (45.2%)
Neutral	5 (10.6%)	5 (12.5%)	6 (14%)	6 (19.4%)
Disagree	2 (4.3%)	3 (7.5%)	1 (2.3%)	1 (3.2%)

© 2021 Patel MS et al. *JAMA Network Open.*
