# Supplementary material for: Effect of Behaviorally Designed Gamification With Social Incentives on Lifestyle Modification Among Adults With Uncontrolled Diabetes: A Randomized Clinical Trial
Source: JAMA Netw Open. 2021 May 24;4(5):e2110255. doi: 10.1001/jamanetworkopen.2021.10255 (PMC8144928; doi:10.1001/jamanetworkopen.2021.10255)
Supplement: Supplement 3. — Data Sharing Statement [file jamanetwopen-e2110255-s003.pdf]

Data Sharing Statement

Patel. Effect of Behaviorally Designed Gamification With Social Incentives on Lifestyle Modification Among Adults With Uncontrolled Diabetes. *JAMA Netw Open*. Published May 24, 2021. doi:10.1001/jamanetworkopen.2021.10255

Data
Data available: Yes
Data types: Deidentified participant data, Data dictionary
How to access data: Harvard Dataverse
When available: With publication

Supporting Documents
Document types: None

Additional Information
Who can access the data: Anyone via Harvard Dataverse
Types of analyses: Any analyses
Mechanisms of data availability: Downloadable from Harvard Dataverse
Any additional restrictions: NA
